# Development and Reorganization of Orientation Representation in the Cat Visual Cortex: Experience-Dependent Synaptic Rewiring in Early Life

**DOI:** 10.3389/fninf.2020.00041

**Published:** 2020-08-20

**Authors:** Shigeru Tanaka, Masanobu Miyashita, Nodoka Wakabayashi, Kazunori O’Hashi, Toshiki Tani, Jérôme Ribot

**Affiliations:** ^1^Center for Neuroscience and Biomedical Engineering, The University of Electro-Communications, Chofu, Japan; ^2^Department of Control and Computer Engineering, National Institute of Technology, Numazu College, Numazu, Japan; ^3^Power Plant Engineering, Engineering & Maintenance Center, All Nippon Airways Co., Ltd., Tokyo, Japan; ^4^Department of Mental Disorder Research, National Institute of Neuroscience, National Center of Neurology and Psychiatry, Kodaira, Japan; ^5^Laboratory for Molecular Analysis of Higher Brain Functions, RIKEN Center for Brain Science, Wako, Japan; ^6^Centre for Interdisciplinary Research in Biology, Collège de France, Paris, France

**Keywords:** orientation maps, self-organization, visual experience, development, sensitive period

## Abstract

To date, numerous mathematical models have been proposed on the basis of some types of Hebbian synaptic learning to account for the activity-dependent development of orientation maps as well as neuronal orientation selectivity. These models successfully reproduced orientation map-like spatial patterns. Nevertheless, we still have questions: (1) How does synaptic rewiring occur in the visual cortex during the formation of orderly orientation maps in early life? (2) How does visual experience contribute to the maturation of orientation selectivity of visual cortical neurons and reorganize orientation maps? (3) How does the sensitive period for orientation plasticity end? In this study, we performed animal experiments and mathematical modeling to understand the mechanisms underlying synaptic rewiring for experience-dependent formation and reorganization of orientation maps. At first, we visualized orientation maps from the intrinsic signal optical imaging in area 17 of kittens reared under single-orientation exposure through cylindrical-lens-fitted goggles. The experiments revealed that the degree of expansion of cortical domains representing the experienced orientation depends on the age at which the single-orientation exposure starts. As a result, we obtained the sensitive period profile for orientation plasticity. Next, we refined our previously proposed mathematical model for the activity-dependent self-organization of thalamo-cortical inputs on the assumption that rewiring is caused by the competitive interactions among transient synaptic contacts on the same dendritic spine. Although various kinds of molecules have been reported to be involved in such interactions, we attempt to build a mathematical model to describe synaptic rewiring focusing on brain-derived neurotrophic factor (BDNF) and its related molecules. Performing computer simulations based on the refined model, we successfully reproduced orientation maps reorganized in kittens reared under single-orientation exposure as well as normal visual experience. We also reproduced the experimentally obtained sensitive period profile for orientation plasticity. The excellent agreement between experimental observations and theoretical reproductions suggests that the BDNF-induced competitive interaction among synaptic contacts from different axons on the same spine is an important factor for the experience-dependent formation and reorganization of orientation selectivity and orientation maps.

## Introduction

In the visual cortices of cats and macaques, there are representations of specific visual features, such as orientation preference, direction of motion preference, ocular dominance, and retinotopy (Hubel and Wiesel, 1962, 1968, 1974, 1977; Hubel et al., 1977; Tusa et al., 1978, 1979; Swindale et al., 1987; Swindale, 1988). These feature representations emerge as columns in the 3D visual cortex, because neuronal response properties are similar in the depth direction from the pia mater to white matter. In particular, the tangential patterns of the columns along the cortical surface are called functional maps, such as orientation maps and ocular dominance maps (Bonhoeffer and Grinvald, 1991; Kim et al., 1999). The development of ocular dominance has been studied in depth because of the relative ease of visual experience manipulations in experiments. In macaques and cats, numerous experiments have shown that monocular deprivation in young animals induced the expansion of cortical domains receiving non-deprived eye inputs and the shrinkage of domains receiving deprived eye inputs (Hubel and Wiesel, 1965; Hubel et al., 1977; Shatz and Stryker, 1978; LeVay et al., 1980; Olson and Freeman, 1980). Even for the rodent visual cortex, it has been revealed that neurons in the binocular zone in the primary visual cortex decreased responses to visual stimuli presented to the deprived eye (Fagiolini et al., 1994; Bear and Rittenhouse, 1999; Fagiolini and Hensch, 2000). In contrast, the experimental manipulation of orientation experience is more difficult than the manipulation needed for the induction of ocular dominance shift. For example, Blakemore and Cooper (1970) exposed kittens to a vertical or horizontal orientation for several hours a day, keeping the kittens inside a drum where vertical or horizontal lines were painted on the inner wall. At other times, they reared the animals in a dark room with their mother cats to prevent visual experience other than vertical or horizontal lines. They demonstrated that neurons selectively responding to experienced orientations were more frequently encountered in their electrophysiological recordings conducted after the visual experience manipulation for several months (Blakemore and Cooper, 1970; Hirsch and Spinelli, 1970). Later, Stryker and Sherk (1975) repeated similar experiments but they were not able to reproduce Blakemore and Cooper’s results with statistical significance.

So far, a few groups have conducted intrinsic signal optical imaging in the cat visual cortex to elucidate the effect of single-orientation exposure on orientation maps, and showed the expansion of cortical territory representing the exposed orientation (Sengpiel et al., 1999; Cloherty et al., 2016). However, in the previous studies, daily dark rearing periods were intervened between single-orientation exposure for several hours a day using either a drum or goggles, so that kittens were cared by their mother cats in dark rooms without exposure to orientations other than the intended orientation (Blakemore and Cooper, 1970; Hirsch and Spinelli, 1970; Rauschecker and Singer, 1981; Sengpiel et al., 1999; Cloherty et al., 2016). To get rid of the dark rearing periods and expose animals to a single orientation stably in the standard animal cages with their mother cats, we have fabricated cylindrical-lens-fitted goggles and developed a method to mount them on the foreheads of kittens (Tanaka et al., 2007). Using these goggles, we have successfully realized continuous single-orientation exposure for a few months without daily dark rearing intervention. Owing to this visual experience manipulation method and using intrinsic signal optical imaging, we have observed the conspicuous expansion of cortical domains representing the exposed orientation (Tanaka et al., 2006), and obtained the sensitive period profile for orientation plasticity in area 17 of kittens, in which 2-week goggle rearing (GR) started from postnatal day 10 (P10) to P73 (Tanaka et al., 2009).

To elucidate biologically plausible mechanisms of synaptic rewiring underlying the map reorganization, we focused on the effects of brain-derived neurotrophic factor (BDNF) and its functionally related molecules on structural plasticity of synaptic connections. We refined our previously proposed model of the self-organization of afferent inputs (Tanaka et al., 2004; Tanaka and Miyashita, 2009), postulating that postsynaptic dendritic spines, presynaptic axonal terminals, and astrocytic processes interact with each other for the formation and elimination of synaptic contacts during development. We applied this model to the development of afferent inputs from the lateral geniculate nucleus (LGN) to the primary visual cortex to examine whether the activity-dependent synaptic rewiring accounts for our experimental observations. At first, we generated random afferent inputs with rough retinotopy. Then, we performed simulations presenting 24 directional drifting gratings to the model retina (12-orientation exposure) for different simulation steps. Next, we resumed simulations presenting only a vertically oriented grating (single-orientation exposure) for a fixed number of simulation steps, using afferent input patterns self-organized under 12-orientation exposure for certain simulation steps. As a result, cortical neurons selectively responding to the exposed vertical orientation increased in number. However, the longer the model experienced gratings from all 12 orientations before only a single orientation was presented, the fewer neurons became selective for this orientation. On the basis of the simulation results, we obtained a theoretical profile of the sensitive period for orientation plasticity, which resembled the experimentally observed one. The excellent agreement between experimental findings and simulation results may shed light on the molecular mechanisms underlying synaptic rewiring in the developing visual cortex.

## Materials and Methods

### Experiments on Cats

The surgical operation and optical imaging were approved by the Institutional Animal Research Committee in RIKEN (No. H13-B040 and H17-2B043) and were performed in accordance with the “*Guiding Principles for the Care and Use of Animals in the Field of Physiological Science*” of the Japanese Physiological Society.

#### Goggles for Single-Orientation Exposure

**FIGURE 1:**
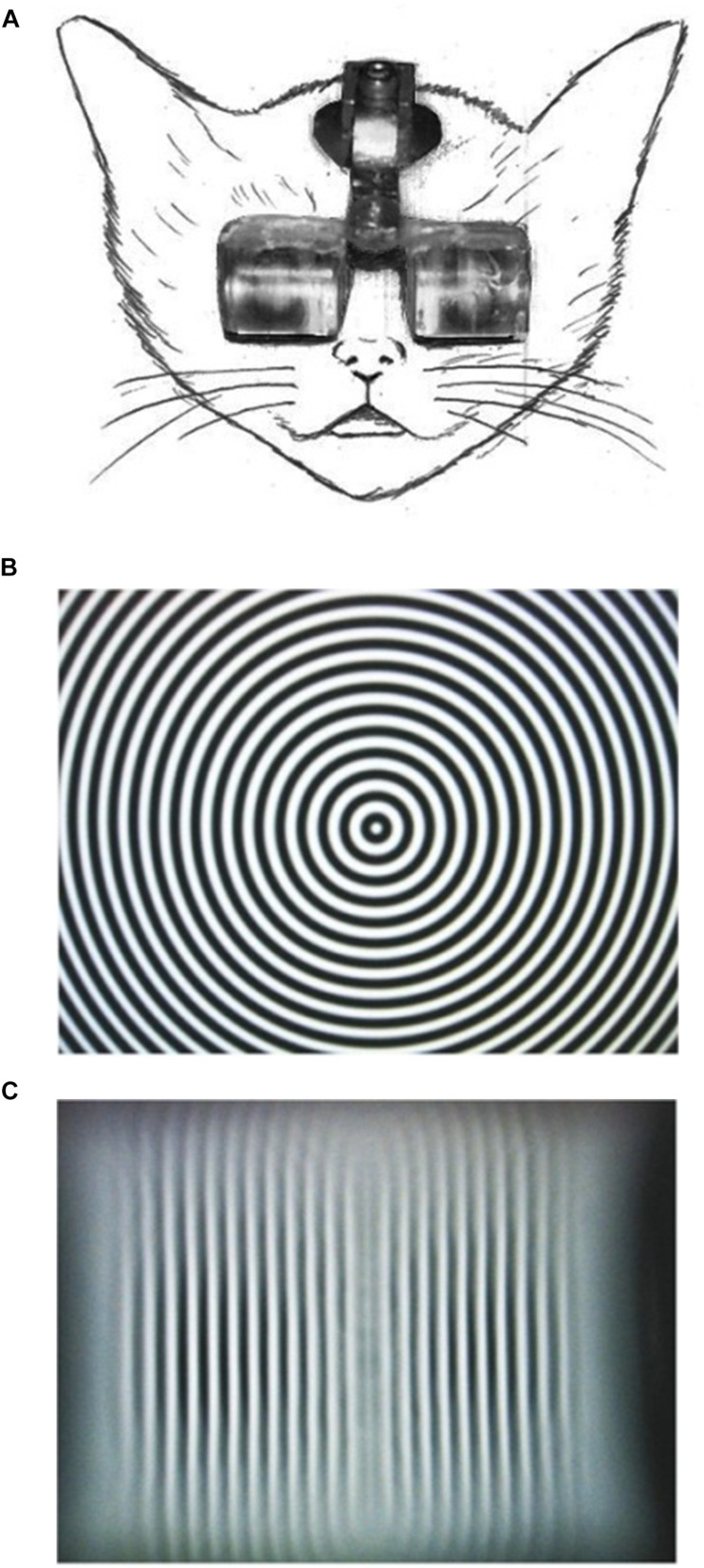
Picture of a goggle-mounted kitten **(A)**, and transformation of a circular stripe **(B)** to a vertical stripe **(C)** by viewing through the goggles.

To realize continuous single-orientation exposure without the intervention of daily dark rearing periods and stabilize the experienced orientation in the retinal coordinate, we fabricated goggles fitted with planoconvex acrylic cylindrical lenses (Figure 1A; lens thickness, 10.0 mm; lens aperture diameter, 15.0 mm; lens power, +67 D), through which the animals experienced elongated visual images of their environments (Figures 1B,C; Tanaka et al., 2007). The goggles were easily attached to and detached from the head holder fixed on the foreheads of kittens (Figure 1A), which enabled us to clean the lenses within a few minutes every day. Using this method, we were able to continuously expose kittens to a single orientation for 4 months in the standard animal cages. The behavior of goggle-mounted kittens was generally not distinct from that of normally reared kittens. We carried out intrinsic signal optical imaging of neural activities in areas 17 and 18 of kittens reared with/without cylindrical-lens-fitted goggles under a normal visual environment to observe how orientation maps are reorganized by single-orientation exposure. The use of cylindrical-lens-fitted goggles is an excellent rearing regimen for single-orientation exposure, which is comparable to monocular deprivation to study the mechanisms of ocular dominance shift. Our intrinsic signal optical imaging showed a marked over-representation of the experienced orientation for any exposed orientation (Tanaka et al., 2006). Surgical operations and optical imaging methods are described elsewhere in detail (Tanaka et al., 2006, 2009), but the basic experimental procedures are shown below.

#### Surgical Procedure

**FIGURE 2:**
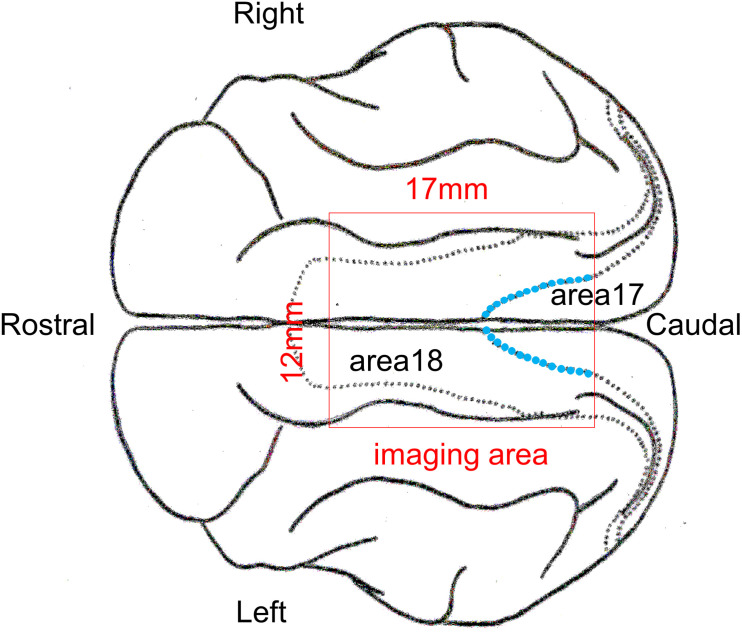
Schematic picture of a cat cerebral cortex in the top view. The optical imaging was performed inside the red rectangle. The anatomically identified border between areas 17 and 18 in each hemisphere inside the imaging area is indicated by the blue dotted curve.

Initial anesthesia was induced using ketamine hydrochloride (5.0 mg/kg, i.m.) following sedation with medetomidine hydrochloride (0.1 mg/kg, i.m.). The animals were fixed on a stereotaxic apparatus and were artificially ventilated with a 60:40% mixture of N_2_O and O_2_ containing 0.5–1.0% isoflurane. Heart rate, end-tidal CO_2_ concentration, and rectal temperature were continuously monitored during surgery. A metal head holder for fixing the goggles and a metal chamber for optical imaging were cemented on the animal’s skull using dental resin, and the skull and dura mater covering the recording area of the lateral gyrus were removed. The cranial window (17 mm × 12 mm) was positioned approximately from P5 to A12 on the AP axis, spanning the midline (Figure 2). Next, the chamber was filled with 2% agar and sealed with a polyvinylidene chloride thin film and a plastic plate. Finally, the frame of the goggles was fixed to a head holder, and the position of the goggles was calibrated so that the cylindrical lenses covered the visual field as widely as possible.

#### Optical Imaging

Animals were anesthetized as in surgery and paralyzed with pancuronium bromide (0.1 mg/kg/h). Then, goggles were taken off for optical imaging in goggle-reared kittens. The animals were artificially ventilated. Contact lenses with appropriate curvatures were used to prevent the drying of the eyes. The cortex was illuminated with a 700-nm wavelength light. The focal plane was adjusted to 500 μm below the cortical surface using a tandem-lens macroscope arrangement (Bonhoeffer and Grinvald, 1996). Intrinsic optical signals were measured while the animals were exposed to visual stimuli displayed on a 20-inch CRT monitor placed 30 cm in front of the animal. Images were obtained with a CCD video camera, and digitized and stored using CAPOS (320 × 240 pixels) (Tsunoda et al., 2001) or Imager 3001 (744 × 480 pixels) (Optical imaging Inc. New York). For each stimulus presentation, the intrinsic signal was recorded for 1.0 s before and 5.0 s after the stimulus onset. A blank stimulus (a uniform gray stimulus) was presented for 15 s between successive captures of intrinsic signals. Each visual stimulus was presented once in a pseudorandom sequence in a single trial of recordings. Twenty-six to 30 trials were collected in each recording session. As visual stimuli, we used full-screen square-wave gratings, which drifted in two directions at six equally spaced orientations (30° interval). In the cat visual cortex, orientation maps appeared in area 18 as well as in area 17. It has been reported that spatial frequencies of 0.5 and 0.15 cpd are optimal for neuronal activation in areas 17 and 18, respectively (Movshon et al., 1978; Bonhoeffer and Grinvald, 1991; Ohki et al., 2000). Thus, we used these two spatial frequencies of grating stimuli to identify areas 17 and 18 differentially. The temporal frequency of the gratings was fixed at 2.0 Hz. The optical imaging in one session was completed within 5 h.

#### Analysis Methods of Intrinsic Signals

One trial of imaging was composed of six frames (duration of each frame, 1 s). To extract stimulus-related intrinsic signals, we subtracted signals recorded in the first frame (without stimulus presentation) from those signals recorded in succeeding frames with stimulus presentations. Then, we averaged the subtracted signals over the 4th to 6th frames for each trial. Next, we applied the generalized indicator function method (GIFM) to these averaged signals (Yokoo et al., 2001), which efficiently excluded noisy signals originating from volume and oxygenation changes in thick blood vessels and spatially slowly varying fluctuations of signals inherent in the recorded intrinsic signals. The GIFM eliminates spatially slowly varying point-spread components of intrinsic signal (Gilbert et al., 1996), which may partially contain responses to the exposed orientation. Therefore, the image data processing based on the GIFM underestimates the degree of over-representation of exposed orientation induced by GR.

Then, we summed the stimulus-related signals over all trials for each stimulus orientation and applied Gaussian low-pass filtering with a 150-mm standard deviation to eliminate high-frequency noise. In this way, we constructed a single-orientation map for each stimulus orientation. We also defined an integrated response-strength map by summing single-orientation maps over all stimulus orientations.

The preferred orientation and orientation magnitude at each pixel were determined by the vector sum method (Bonhoeffer and Grinvald, 1991). The orientation magnitude, which is the modulation amplitude of the second harmonic components in the Fourier expansion of signals with respect to the stimulus orientation, was used as one of indices for orientation selectivity. The orientation polar map was constructed with the preferred orientation and orientation magnitude as color and brightness, respectively.

Area 17 was identified to be the cortical domains, in which the integrated response strength for the stimulus spatial frequency of 0.5 cpd is larger than a half of those averaged inside the recorded area. In orientation polar maps, we delimited such functionally identified area 17 by the white lines. Although the orientation selectivity of neurons in the two areas seems to develop almost in parallel, we focused on the development and reorganization of orientation maps in area 17 in this paper (Supplementary Figure 1).

### Mathematical Modeling

#### Hypothetical Mechanisms of Synaptic Rewiring

**FIGURE 3:**
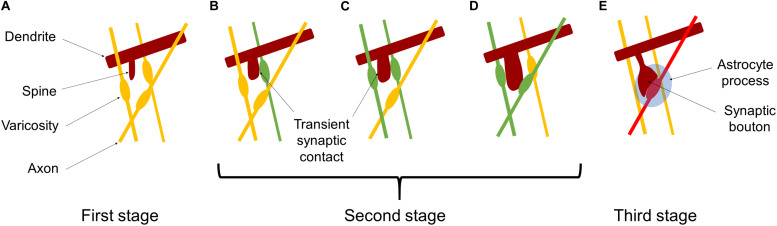
Schematic pictures of synaptic rewiring. **(A)** A spine (brown) is generated and elongated toward adjacent axonal varicosities (orange). **(B)** The spine catches one of axonal varicosities to make a transient synaptic contact (green). **(C)** The spine changes its shape and moves to make another transient synaptic contact (green), and the two synaptic contacts start to compete each other. **(D)** One of the transient synapses survives on the spine (green) and the other retracts (orange). On the other hand, the spine makes a transient synaptic contact with another axon to start competition again. **(E)** In the late stage of the sensitive period, one transient synaptic contact wins the competition as a stabilized synapse (red). Pre- and postsynaptic structures are wrapped by the astrocyte process, forming a tripartite synapse.

For building a mathematical model of synaptic rewiring during development, our basic idea is that individual synapses compete for survival, and only functionally adequate synapses can survive. Such synapse competition may be mediated and/or modulated by interactions among various types of molecules, such as cell adhesion molecules, chondroitin sulfate proteoglycans, neurotrophins, and proteases. These molecules and functional proteins, which are expressed on the cell surface or released to the extracellular space, may work to stabilize labile synaptic contacts in some cases and destabilize synapses in some other cases. Three stages of synapse maturation are schematically shown in Figure 3. At the first stage (Figure 3A), a neurite (brown) generated on a dendrite and elongated toward neighboring varicosities on presynaptic axons (yellow). At the second stage, one of the varicosities wins the competition to make a transient synaptic contact (green), as shown in Figure 3B. Then, another axonal varicosity makes a transient synaptic contact (green), and the two synaptic contacts start competing to occupy the spine, as shown in Figure 3C. One of the transient synaptic contacts survives (green) on the spine and the other retracts (yellow), as depicted in Figure 3D. Such a rewiring process of transient synaptic contacts is repeated during the sensitive period (Figures 3B–D). At the third stage, transient and labile synaptic contacts disappear leaving one stabilized synapse (red). Finally pre- and postsynaptic structures are wrapped by the astrocyte process, forming a tripartite synapse (Araque et al., 1999), as shown in Figure 3E.

To mathematically describe synapse competition that may occur on small regions of dendrites, resulting in the stabilization and elimination of transient synapses, here we postulate the following Lotka-Volterra equations (Hofbauer and Sigmund, 1988; Fukai and Tanaka, 1997) in an analogy to ecological systems in which species compete with each other for the same resources and/or niches. When ρ_*k*_ represents the population of *k*th species, the dynamics of {ρ_*k*_} is given by:

(1) $$\frac{d}{d\,t}\,\rho_{k}=a_{k}\,\rho_{k}-\sum_{k^{\prime }}\rho_{k^{\prime }}\,\rho_{k}$$

where *a*_*k*_ describes the effect of common resources on the growth rate of the *k*th species population. The second term represents competition among species. According to the basic property of the competitive Lotka–Volterra equation, a winner of the competition is determined to be a non-zero element of a stable fixed point solution to Equation (1). When *a*_*k_0_*_ is the maximum of all *a*_*k*_′ s, that is,

(2) $$k_{0}=\arg\,\max_{k}\left\{a_{k}\right\}$$

the steady-state solution is given by

(3) $$\rho_{k_{0}}=a_{k_{0}}\;\text{and}\;\rho_{k}=0\left(\text{for}k\neq k_{0}\right)$$

For synapse formation, *a*_*k*_ corresponds to the growth rate of synaptic contacts at a small region of a target dendrite, mediated by various types of cell adhesion molecules and neurotrophins; some molecules are secreted in an activity-dependent manner. Among them, we pay a special attention to the effects of the BDNF (Binder and Scharfman, 2004) and tissue-plasminogen activator (tPA) (Mataga et al., 2002, 2004).

BDNF regulates the late-phase long-term potentiation (L-LTP) of synaptic transmission efficacy (Frey et al., 1996) and facilitates survival of synapses in the developing brain (Cunha et al., 2010; Numakawa et al., 2010). BDNF is synthesized as pre-proBDNF, and its presequence is cleaved off in the endoplasmic reticulum. Then, proBDNF is sorted in the Golgi apparatus into the regulated pathway and constitutive pathway (Kuczewski et al., 2009). In these pathways, proBDNF is considered to be partially converted to mature BDNF (mBDNF), and proBDNF and mBDNF are contained inside vesicles. In the regulated pathway, they are released to the extracellular space by Ca^2+^-induced exocytosis. Released proBDNF and mBDNF bind to pan-neurotrophin receptor p75 and TrkB receptors, respectively (Yang et al., 2016), which are expressed on the cell surface of neurons and glial cells. During development, proBDNF signaling through p75^NTR^ leads to the elimination of transient synaptic contacts (Teng et al., 2005; Singh et al., 2008), and the binding of mBDNF to TrkB leads to L-LTP (Pang et al., 2004) and stabilizes transient synaptic contacts. Particularly, in neuromuscular junctions, synaptic retraction has been reported to be mediated by presynaptic p75^NTR^ signaling during synaptic competition (Je et al., 2012). In the rat visual cortex, p75^NTR^ expression in parvalbumin-positive (PV) cells and putative pyramidal neurons is likely to be temporally regulated during development (Bracken and Turrigiano, 2009), and decreases between P14 and P26, at a time when PV cell synapse numbers increase dramatically (Baho et al., 2019).

On the other hand, tPA is known to be a protease that cleaves plasminogen to generate plasmin (Tsai, 2017). In turn, plasmin cleaves perineuronal nets composed of a condensed matrix of chondroitin sulfate proteoglycans (Berardi and Pizzorusso, 2004; McRae and Porter, 2012), and converts proBDNF to mBDNF in the extracellular space. In addition, it has been reported that the secretion of tPA from neurons is also triggered by intracellular calcium concentration elevated by neuronal activity. Importantly, for a higher elevation of Ca^2+^ concentration, which occurs as in tetanic stimulation, tPA is released to activate the tPA/plasmin system, and the resultant plasmin converts proBDNF to further increase the amount of mBDNF in the extracellular space (Greenberg et al., 2009; Nagappan et al., 2009). This results in the induction of L-LTP and the stabilization of transient synaptic contacts. In contrast, a moderate elevation of Ca^2+^ concentration releases less tPA, which is unlikely to activate the tPA/plasmin system. A higher ratio of the amount of proBDNF to that of mBDNF in the synaptic cleft induces LTD and consequently elimination of transient synapses. On the basis of these facts, we hypothesize that a high concentration of intracellular Ca^2+^ taken up through NMDA channels and/or voltage-dependent calcium channels tends to assist the survival and stabilization of synapses. A moderate Ca^2+^ concentration tends to facilitate synapse pruning.

Brain-derived neurotrophic factor is also secreted from the constitutive pathway in an activity-independent manner (Lesmann and Brigadski, 2009; Cunha et al., 2010). The release of BDNF from the constitutive pathway and/or from presynaptic axons may contribute to the attractive interaction between dendritic spines and varicosities along the axons at the first and second stages of synaptogenesis (Figures 3A–D). BDNF released from postsynaptic dendrites may also induces competition of a presynaptic contact with other contacts (Figures 3B–D). At the late stage (Figure 3E), the expression of p75^NTR^ on the surfaces of pre- and postsynaptic neurons increases to bind proBDNF, resulting in the weakening of synaptic contacts. Instead, processes of a neighboring astrocyte expand contact area with the dendrite. The competition between the synaptic contacts and the processes of the astrocyte leads to the morphological changes of the dendritic spine, and finally the formation of stable tripartite synapses.

Thus, we propose the following dynamics of the synaptic contact areaρ_*k*_ at the second stage:

(4) $$\tau_{s\,y\,n}\,\frac{d}{d\,t}\,\rho_{k}=\left(a_{k}+b_{k}\right)\,\rho_{k}-\sum_{k^{\prime }}\rho_{k^{\prime }}\,\rho_{k}$$

(5) $$a_{k}=\bar{a}+\Delta \,a_{k}$$

(6) $$b_{k}=b_{k}^{m\,a\,t\,u\,r\,e}-b_{k}^{p\,r\,o}$$

*a*_*k*_ represents the activity-independent synapse growth rate, which is assumed to be proportional to the amount of cell adhesion molecules and BDNF secreted from the constitutive pathway. Owing to its activity independence, *a*_*k*_is regarded to be constant over time. However, it may vary for different locations, because different amounts of cell adhesion molecules may be expressed at different synaptic contacts. The mean and fluctuation of *a*_*k*_ are denoted as $\bar{a}$ andΔ*a*_*k*_, respectively. *b*_*k*_represents the Ca^2+^-dependent synapse growth rate, which is given by the difference in the amounts of mBDNF and proBDNF, $b_{k}^{m\,a\,t\,u\,r\,e}-b_{k}^{p\,r\,o}$. τ_*syn*_is a constant related to the time scale of synaptic modification. In early life, in which the perineuronal net is labile, τ_*syn*_is small and synaptic contacts are easy to change, whereas after the perineuronal net is robustly established, τ_*syn*_is sufficiently large and synaptic connections no longer change. Without loss of generality, we set $\bar{a}=1$.

A higher elevation of Ca^2+^ concentration is likely to occur when NMDA receptor activation coincides with backpropagating action potential (bAP) (Markram et al., 1997; Koester and Sakmann, 1998, 2000; Nevian and Sakmann, 2004) and triggers a Ca^2+^-dependent dendritic release of tPA as well as BDNF. The released BDNF is cleaved by tPA in the extracellular space to produce mBDNF. In contrast, a moderate elevation of Ca^2+^ concentration may take place when only NMDA receptors are activated in the absence of bAPs. In this case, the proBDNF may be richer in the released BDNF, because the tPA/plasmin system is less activated. Consequently, *b*_*k*_ at the *k*th synaptic contact is obtained as

(7) $$b_{k}=\Theta \,\left(\varsigma^{p\,o\,s\,t}-\vartheta \right)\,\varsigma^{p\,o\,s\,t}\,\eta_{k}^{p\,r\,e}-c^{D/P}\,\left(1-\Theta \,\left(\varsigma^{p\,o\,s\,t}-\vartheta \right)\right)\,\varsigma^{p\,o\,s\,t}\,\eta_{k}^{p\,r\,e}$$

Here, ς*^post^* andϑ are postsynaptic membrane depolarization and the threshold for the generation of action potential, respectively. $\eta_{k}^{p\,r\,e}$ represents firing frequency transmitted through the *k*th synaptic contact. Θ(*x*) is the Heaviside function of *x*. Θ(ς*^post^*−ϑ) takes unity for the arrival of a bAP generated at the cell body (ς*^post^* > ϑ), and 0 otherwise. Therefore, the first term on the right-hand side of Equation (7) represents the amount of mBDNF that facilitates L-LTP induction. In contrast, the second term represents the amount of proBDNF that facilitates LTD. Here, 1−Θ(ς*^post^*−ϑ) indicates the absence of bAPs. The coefficient *c*^*D*/*P*^ is the efficiency ratio of depression to potentiation. The basic logic mentioned above is common to that of the BCM model (Bienenstock et al., 1982). However, the present model is devoted to the rewiring of synaptic contacts rather than synaptic efficacy changes at individual synapses. The synaptic efficacy at the *k*th axonal bouton changes depending on *b*_*k*_ alone: LTP occurs for *b*_*k*_ > 0, whereas LTD occurs for *b*_*k*_ < 0. The rewiring of synaptic contacts from the *k*th axonal bouton to the *k’*th axonal bouton is determined by the difference of the growth rates $b_{k^{\prime }}-b_{k}$, as described in the following section.

Neuronal activity changes in the order of milliseconds, but synaptic contacts change in longer time scales τ_*syn*_. Therefore, the dynamics of synaptic contacts can be approximately described by an equation in which *b*_*k*_ is replaced with its temporal average ⟨*b*_*k*_⟩.

#### Activity-Dependent Synaptic Rewiring

The present model focuses on synaptic competitive rewiring repeatedly occurs during the second stage (Figures 3B–D). It is considered that a transient synaptic contact receiving a larger amount of mBDNF and a less amount of proBDNF is likely to win the competition against the other transient synaptic contacts. More specifically, when there are two transient synaptic contacts from the *k*th and *k’*th axons to the spine in question, for $\left\langle b_{k^{\prime }}\right\rangle >\left\langle b_{k}\right\rangle$ *k’*th synaptic contact is stabilized to survive, and the *k*th contact is disconnected. Then, the spine changes its morphology to make a transient contact with another adjacent axon as a next competitor. After repeated rewiring of transient synaptic contacts, adequate synapses are formed at suitable locations of a target neuron.

Here, we rewrite a steady-state solution to Equation (3) with respect to {ρ_*k*_} as {σ_*k*_|σ_*k*_ = 1,0;∑_*k*_σ_*k*_ = 1}, omitting the absolute value that may represent synaptic transmission efficacy. The mathematical expression σ_*k*_ = 1 or 0 indicates the presence or absence of a synaptic connection. This approximation is based on the idea that activity propagation during development depends more on the variation in the number of synaptic connections than the variation in the transmission efficacy at each synapse. The probability of synaptic rewiring from $\left\{\sigma_{k}=1,\sigma_{k^{\prime }}=0\right\}$ to $\left\{\sigma_{k}=0,\sigma_{k^{\prime }}=1\right\}$ is given by the ensemble-averaged Heaviside function of the difference in the synapse growth rates as follows:

(8) $$P\left(\sigma_{k}=1\to \sigma_{k^{\prime }}=1\right)={\left[\Theta \left(\left\langle b_{k^{\prime }}\right\rangle +\Delta a_{k^{\prime }}-\left\langle b_{k}\right\rangle -\Delta a_{k}\right)\right]}_{a\,v}$$

where [*X*]_*av*_ indicates ensemble averaging of *X* with respect to fluctuation {Δ*a*_*k*_}. When we assume that {Δ*a*_*k*_} obeys the Gaussian distribution with mean 0 and variance *s*^2^ for mathematical simplicity, the ensemble-averaged rewiring probability is given by the error function. The error function is approximated by the logistic function with an excellent fitness (Tanaka, 1990). Finally, the rewiring probability of transient synaptic contacts is obtained as

(9) $$P\left(\sigma_{k}=1\to \sigma_{k^{\prime }}=1\right)=\frac{1}{1+e^{-\beta \,\left(\left\langle b_{k^{\prime }}\right\rangle -\left\langle b_{k}\right\rangle \right)}}$$

Using *s*, the inverse fictitious temperature β in Equation (9) is given as

(10) $$\beta =\frac{2}{\sqrt{\pi }\,s}$$

The spine motility, which is also regulated by the balance in the activation of TrkB and p75^NTR^, affects the accessibility to an adjacent axonal varicosity as a next competitor. BDNF released from the constitutive pathway of a target dendrite and/or released from varicosities along the presynaptic axons may be responsible for the TrkB and p75^NTR^ activation. It is considered that the probability of transition from an axonal varicosity to a transient synaptic contact is given by a similar form of Equation (9) as

(11) $$P\left(\sigma_{k}=0\to \sigma_{k}=1\right)=\frac{1}{1+e^{-\beta \,g_{k}\,\left(t\right)}}$$

where *g*_*k*_(*t*) represents the growth rate of a dendritic spine toward a varicosity on the *k*th axon to make a transient synaptic contact. It is mainly determined by the difference in the activation of TrkB and p75^NTR^ expressed on the spine in question. For simplicity, we consider that BDNF released from the activity-independent constitutive pathway signals to the spine in an autocrine fashion, and that the expression of p75^NTR^ strongly depend on the age: In the middle of the sensitive period, the expression level of p75^NTR^ is sufficiently low, and at the late stage, however, the level of p75^NTR^ increases. To model the effect of the age-dependent expression of p75^NTR^, we adopt the following form of *g*_*k*_(*t*):

(12) $$g_{k}\,\left(t\right)=-\xi_{\max}\,{\left(\frac{t}{T_{p}+t}\right)}^{4}$$

Here,ξ_max_ is proportional to the maximum amount of p75^NTR^ expression on the *k*th axonal bouton around the end of the sensitive period, and *T*_*p*_ is a time constant that determines the width of the time window of synaptic plasticity. For simplicity, ξ_max_ and *T*_*p*_ are assumed to be constant over the primary visual cortex. *g*_*k*_(*t*) should be rapidly increasing and then saturated within the sensitive period. Simulation results suggest that the exponent 4 in Equation (12) well reproduces the sensitive period profile for orientation plasticity.

Now, we apply the mathematical framework derived above to the activity-dependent rewiring of geniculo-cortical afferent inputs for the formation of oriented RFs of individual cortical neurons and the formation of orientation representations. We assumed retinotopic projection from the retina to LGN and the presence of four types of LGN neuron: lagged and non-lagged types (Saul and Humphrey, 1990) for each of the ON- and OFF-center cells. When *k* represents a position of an LGN neuron, and μ_1_ = 1(−1) and μ_2_ = 1(−1) represent ON (OFF)-center and non-lagged (lagged) types, respectively, Equation (7) in this specific application is rewritten as

(13) $$\left\langle b_{i,k,\mu_{1},\mu_{2}}\right\rangle =\left(1+c^{D/P}\right)\,\left\langle \Theta \,\left(\varsigma_{i}^{C\,X}-\vartheta_{i}\right)\,\varsigma_{i}^{C\,X}\,\eta_{k,\mu_{1},\mu_{2}}^{L\,G\,N}\right\rangle -c^{D/P}\,\left\langle \varsigma_{i}^{C\,X}\,\eta_{k,\mu_{1},\mu_{2}}^{L\,G\,N}\right\rangle$$

$\varsigma_{i}^{C\,X}\,\text{and}\,\eta_{i}^{C\,X}$ represent the membrane potential and firing rate of the *i* th cortical neuron, respectively. $\eta_{k,\mu_{1},\mu_{2}}^{L\,G\,N}$ is the firing rate of the LGN neuron specified by cell type μ_1_andμ_2_ and retinotopic position *k*. Here, we assume that electrotonic distance is sufficiently long beyond the dendritic extent of a postsynaptic neuron, and the evoked membrane potential is independent of the location of a synaptic input on the dendrite. The membrane potential of a cortical neuron $\varsigma_{i}^{C\,X}$ measured from the resting level is given by the sum of the afferent input of LGN neurons’ activities $\varsigma_{i}^{A\,F\,F}$ and the lateral interaction:

(14) $$\varsigma_{i}^{C\,X}=\varsigma_{i}^{A\,F\,F}+\sum_{i^{\prime }\neq i}U_{i,i^{\prime }}\,\eta_{i^{\prime }}^{C\,X}=v\,\sum_{k,\mu_{1},\mu_{2}}n_{i,k,\mu_{1},\mu_{2}}\,\eta_{k,\mu_{1},\mu_{2}}^{L\,G\,N}+\sum_{i^{\prime }\neq i}U_{i,i^{\prime }}\,\eta_{i^{\prime }}^{C\,X}$$

where *n*_*i,k,μ_1_,μ_2_*_ is the total number of synaptic connections from a presynaptic neuron (*k*,μ_1_,μ_2_) to different locations on the dendrites of postsynaptic neuron *i*:

(15) $$n_{i,k,\mu_{1},\mu_{2}}=\sum_{j}\sigma_{i,j,k,\mu_{1},\mu_{2}}$$

Using the function *F*(*x*) = *x*Θ(*x*), the firing rate of the postsynaptic neuron is half-rectified as

(16) $$\eta_{i}^{C\,X}=F\,\left(\varsigma_{i}^{C\,X}-\vartheta_{i}\right)$$

The threshold ϑ_*i*_ at the *i*th neuron is assumed to be the long-time average of the membrane potential $\varsigma {}_{i}^{C\,X}$.

The function *U*_*i,i’*_ represents short-range excitatory and long-range inhibitory lateral connections between model cortical neurons *i* and *i*′ given as

(17) $$U_{i,i^{\prime }}=q\,\left(\frac{1}{2\,\pi \,\lambda_{e\,x}^{2}}\,e^{-\frac{{\left({\vec{x}}_{i}-{\vec{x}}_{i^{\prime }}\right)}^{2}}{2\,\lambda_{e\,x}^{2}}}-\frac{\kappa_{i\,n\,h/e\,x}}{2\,\pi \,\lambda_{i\,n\,h}^{2}}\,e^{-\frac{{\left({\vec{x}}_{i}-{\vec{x}}_{i^{\prime }}\right)}^{2}}{2\,\lambda_{i\,n\,h}^{2}}}\right)$$

${\vec{x}}_{i}$ indicates the position of neuron *i* in the model visual cortex. The parameters λ_*ex*_ and λ_*inh*_ represent the extent of excitatory and inhibitory lateral connections, respectively. *q* and κ_*inh/ex*_ are the strength of lateral interaction and the ratio of the strengths of inhibition to excitation, respectively.

**FIGURE 4:**
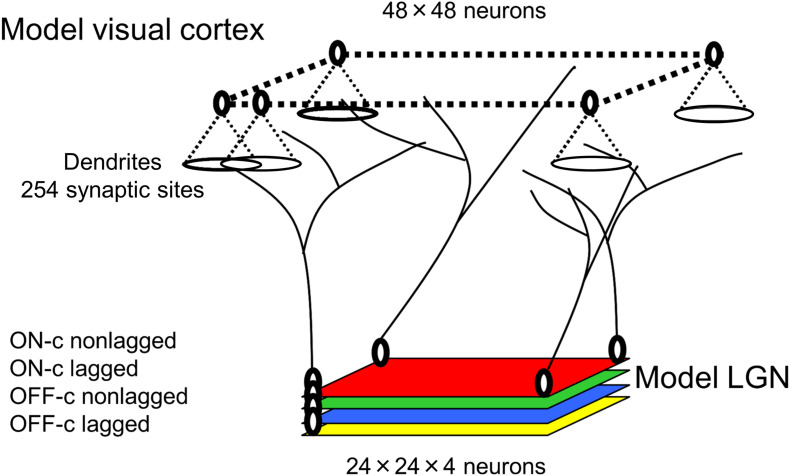
Model visual pathways and layer configurations.

A schematic diagram of visual pathways in our model is shown in Figure 4. In simulations for the development of visual cortical neurons under normal visual conditions, model LGN neurons are activated by the balanced presentation of grating stimuli drifting in 24 directions. In simulations for the development under the exposure to a single orientation through cylindrical-lens-fitted goggles, model LGN neurons are activated by the presentation of only a vertically oriented grating drifting leftward or rightward.

#### Activities of Model LGN Neurons

The reverse correlation method of electrophysiology has revealed that LGN neurons have spatiotemporally separable ON- or OFF-center RFs (DeAngelis et al., 1995). We use the following mathematical expressions of the LGN RFs, assuming the difference of Gaussian functions as spatial components, and the products of exponential and trigonometric functions as temporal components:

(18) $$R_{k,\mu_{1},\mu_{2};l}^{L\,G\,N}\,\left(t\right)=S_{k,\mu_{1};l}\,T_{\mu_{2}}\,\left(t\right)$$

Here, the spatial component *S*_*k,μ_1_;l*_ and temporal one *T*_μ2_(*t*) are given, respectively, as

(19) $$S_{k,\mu_{1};l}=\mu_{1}\,\left[\frac{1}{2\,\pi \,\lambda_{c}^{2}}\,\exp\left(-\frac{d_{k,l}^{2}}{2\,\lambda_{c}^{2}}\right)-\frac{\kappa_{s/c}}{2\,\pi \,\lambda_{s}^{2}}\,\exp\left(-\frac{d_{k,l}^{2}}{2\,\lambda_{s}^{2}}\right)\right]$$

(20a) $${\text{T}}_{+1}\,\left(t\right)=A\,\exp\left(-\frac{t}{\tau_{d\,e\,c\,a\,y}}\right)\,\sin\left(2\,\pi \,f_{R\,F\,f\,r\,e\,q}\,t\right)\,\Theta \,\left(t\right)\,\Theta \,\left(T_{w\,i\,n\,d\,o\,w}-t\right)$$

(20b) $${\text{T}}_{-1}\,\left(t\right)=-A\,\exp\left(-\frac{T_{w\,i\,n\,d\,o\,w}+\tau_{l\,a\,t\,e\,n\,c\,y}-t}{\tau_{d\,e\,c\,a\,y}}\right)$$

$$\times \sin\left[2\,\pi \,f_{R\,F\,f\,r\,e\,q}\,\left(t-\tau_{l\,a\,t\,e\,n\,c\,y}\right)\right]$$

$$\times \Theta \,\left(t-\tau_{l\,a\,t\,e\,n\,c\,y}\right)\,\Theta \,\left(T_{w\,i\,n\,d\,o\,w}+\tau_{l\,a\,t\,e\,n\,c\,y}-t\right)$$

Parameters λ_*c*_ and λ_*S*_ indicate the extents of the center and surround subfields of LGN neurons, respectively. κ_*s/c*_is the ratio of the strength of the response to a surround subfield stimulation to that to a center subfield stimulation. d_*k,l*_ is the distance in the visual field between positions of the RF center of an LGN neuron *k* and a light spot presented at *l*. *T*_*window*_,τ_*latency*_,τ_*decay*_, and *f*_*RFfreq*_ represent the time window of temporal RF, the latency of the lagged response to the non-lagged response, the decay constant of temporal RF, and the temporal frequency of RF, respectively. The values of all parameters in Equations (19), (20a), and (20b) were estimated by the least squares method to fit the experimental data (DeAngelis et al., 1995), and listed in Table 1.

**TABLE 1:** List of parameters and their values.

Parameter	Comments	Value
**Afferent input-induced membrane potential**		
v	Contribution of afferent inputs to membrane potential	0.045
**Cortical interaction function**		
λ_*ex*_	Extent of excitatory interaction function	82 mm
λ_*inh*_	Extent of inhibitory interaction function	328 mm
*q*	Strength of cortical interaction	4.0
κ_*inh*/*ex*_	Inhibition/excitation ratio	1.0
**Model visual cortex**		
*a*^*cell*−*cell*^	Distance between nearest neighbor neurons	50 mm
*a*^*spine*−*spine*^	Distance between nearest neighbor spines	15 mm
**LGN receptive field**		
λ_*c*_	Extent of center subfield	0.225 deg
λ_*s*_	Extent of surround subfield	0.9 deg
κ_*s/c*_	Surround/center ratio	1.0
*A*	Coefficient of RF	1.0
τ_*decay*_	Decay constant of temporal RF	100 ms
*f*_*RFfreq*_	Temporal frequency of RF	5 Hz
*T*_*window*_	Time window of temporal RF	200 ms
τ_*latency*_	Latency of lagged response	57 ms
**Sinusoidal moving grating stimulus**		
*f*_*spat*_	Spatial frequency of gratings	0.4 cpd
*f*_*temp*_	Temporal frequency of gratings	4 Hz
**Transition probability function**		
β	Fictitious inverse temperature	250
*c*^*D*/*P*^	Efficiency ratio of depression to potentiation	2.0
ξ_max_	Maximum amount of p75NTR expression	0.8
*T*_*p*_	Time window that enables synapses to be rewired	15.75 MCs

In simulations, we used a set of sinusoidal moving grating stimuli {*z*_*l*_(*t*|θ_*DR*_,*f*_*spat*_,*f*_*temp*_)}:

(21) $$z_{l}\,\left(t|\theta_{D\,R},f_{s\,p\,a\,t},f_{t\,e\,m\,p}\right)\;=\text{cos}\,\left[2\,\pi \,f_{s\,p\,a\,t}\,\left(x_{l}\,\cos\theta_{D\,R}+y_{l}\,\text{sin}\,\theta_{D\,R}\right)-2\,\pi \,f_{t\,e\,m\,p}\,t\right]$$

where θ_*DR*_, *f*_*spat*_, and *f*_*temp*_ indicate the direction of movement, spatial frequency, and temporal frequency of a stimulus grating, respectively. *x*_*l*_ and *y*_*l*_are the two components of a given position vector of the *l*th pixel in the 2D visual field. Responses of model LGN neurons are given by

(22) $$\eta_{k,\mu_{1},\mu_{2}}^{\text{LGN}}\,\left(t\right)=F\,\left[\sum_{l}\int_{-\infty }^{t}dt^{\prime }\,R_{k,\mu_{1},\mu_{2}}^{\text{LGN}}\,\left(t-t^{\prime }\right)\,z_{l}\,\left(t^{\prime }|\theta_{D\,R},f_{s\,p\,a\,t},f_{t\,e\,m\,p}\right)\right]$$

It is considered that LGN neurons transmit only signals localized around spatial and temporal frequencies characteristic of their receptive fields. Spatial frequencies are band-pass-filtered by the Difference of Gaussian function in Equation (19), and temporal frequencies are also band-pass-filtered by the trigonometric functions in Equations (20a) and (20b). To save time for simulations, we presented only a grating of spatial and temporal frequencies, whose values were close to that of LGN characteristic frequencies. Even if we carry out simulation presenting some range of spatial and temporal frequencies around LGN characteristic frequencies at the expense of time for simulation, we may see cortical neurons strongly responding to narrow ranges of spatial and temporal frequencies. Such simulations remain to be performed in the future study.

More essentially, there is a question about whether patterned visual experience such as grating stimuli in the present model is needed to form orientation selectivity of individual cortical neurons and orderly orientation maps. The formation of orientation-selective receptive fields requires the parallel alignments of receptive field centers of ON- and OFF-center LGN neurons. This requires a specific structure of the activity correlations in three types of LGN neuron pairs: ON-c/ON-c, ON-c/OFF-c, OFF-c/OFF-c. If orientation maps emerge without pattern vision, correlated activities may stem from the LGN or retinal intrinsic circuits. In the present study, we do not address this question, focusing on the issue of experience of drifting oriented gratings on cortical orientation representation.

#### Computer Simulation Methods

All programs coding the algorithms for experience-dependent synaptic rewiring of geniculo-cortical afferent inputs and the analysis of response properties of individual neurons and orientation representation in the model visual cortex are available at https://github.com/slforg/synaptic_rewiring_programs. As shown in Figure 4, our model visual cortex consists of neurons in 48 × 48 square arrangements, each of which has a disk-shaped dendritic field of 254 synaptic sites. The model LGN is composed of four layers of 24 × 24 square arrangements of neurons, where the layers represent ON-center non-lagged, OFF-center non-lagged, ON-center lagged, and OFF-center lagged types (Saul and Humphrey, 1990, 1992). The four types of neuron in the model LGN send axons to the model visual cortex. Before conducting simulations, each cortical neuron receives randomized afferent inputs from 324 (9 × 9 × 4) LGN neurons around a position that retinotopically corresponds to the position of the synaptic site in the model cortex. This randomization process prepares a rough retinotopic projection from the LGN to the visual cortex as an initial pattern of afferent inputs. To avoid the edge effect of the finite model cortex and LGN, we impose periodic boundary conditions on the cortex and LGN. The computer simulation of synaptic rewiring was performed by the Monte Carlo (MC) simulation method (Metropolis et al., 1953).

Let us suppose the synaptic input to originate from the LGN neuron specified by (*k*,μ_1_,μ_2_) and let the selected cortical neuron be the *i* th neuron. At each trial of synaptic update, at first a synaptic site of a cortical neuron is randomly selected. Then, an LGN neuron is randomly picked up from 9 × 9 × 4 LGN neurons, which are retinotopically close to the *i* th cortical neuron, according to the probability given by Equation (11). The values of ⟨*b*_*i*,*k*,μ_1_,μ_2__⟩ and $\left\langle b_{i,k^{\prime },\mu_{1}^{\prime },\mu_{2}^{\prime }}\right\rangle$are calculated using a synaptic input pattern {*n*_*i*,*k*,μ_1_,μ_2__} before and after synaptic update. A current synaptic input (*k*,μ_1_,μ_2_) is replaced with a candidate synaptic input $\left(k^{\prime },\mu_{1}^{\prime },\mu_{2}^{\prime }\right)$ according to the rewiring probability given by Equation (9). Since the MC simulation does not solve the dynamical equations of synaptic inputs, the simulation step does not represent the real time of evolution. Nevertheless, it is regarded to be real time *t* in this study. One MC step is set to be 585216 (48 × 48 × 254) trials of the update of afferent synaptic inputs, which correspond to the total number of synaptic sites. That is, the update of all synaptic sites is attempted on average once for one MC step. In the computer simulation of orientation map formation, there is a critical inverse temperature β_*c*_. For β > β_*c*_, orientation maps emerge, whereas for β < β_*c*_, no orientation maps are formed. In our parameter setting of simulations, β_*c*_≈28 (see Supplementary Figure 2). In the simulations to reproduce the sensitive period profile for orientation plasticity, we assume that orientation map formation starts at *t=0*. In other words, 0 < β < β_*c*_ at *t* < 0 and β > β_*c*_ at *t* > 0. *t=0* may correspond to some postnatal day between P20 and P24.

To calculate the firing rate $\eta_{i}^{C\,X}$of the *i* th cortical neuron using Equations (14) and (16), we need firing rates ${\left\{\eta_{i^{\prime }}^{C\,X}\right\}}_{i^{\prime }\neq i}$ of other cortical neurons to calculate the contribution of the lateral interaction term. This makes it difficult to calculate the growth rate ⟨*b*_*i*,*k*,μ_1_,μ_2__⟩ in Equation (13). To obtain its approximate expression of the growth rate at the *i*th cortical neuron, the contribution of the other neurons’ activities through the lateral connections to the membrane depolarization $\varsigma_{i}^{C\,X}$ is assumed to be sufficiently smaller than the contribution of LGN neurons’ activities through the afferent inputs $\varsigma_{i}^{A\,F\,F}$. We expand $\eta_{i}^{C\,X}$and ⟨*b*_*i*,*k*,μ_1_,μ_2__⟩ with respect to the lateral interaction, and neglect terms higher than the first order. We also omit the delta function appearing as the derivative of the Heaviside function, because in the discrete numerical calculation, the argument of the delta function rarely become exact zero. Thus, we obtain the following expressions:

(16’) $$\eta_{i}^{C\,X}\approx \eta_{i}^{A\,F\,F}+\Theta \,\left(\varsigma_{i}^{A\,F\,F}-\vartheta_{i}\right)\,\sum_{i^{\prime }\neq i}U_{i,i^{\prime }}\,\eta_{i^{\prime }}^{A\,F\,F}$$

$$\left\langle b_{i,k,\mu_{1},\mu_{2}}\right\rangle \approx \left(1+c^{D/P}\right)\,\left[\left\langle \eta_{i}^{A\,F\,F}\,\eta_{k,\mu_{1},\mu_{2}}^{L\,G\,N}\right\rangle +\vartheta \,\left\langle \Theta \,\left(\varsigma_{i}^{A\,F\,F}-\vartheta_{i}\right)\,\eta_{k,\mu_{1},\mu_{2}}^{L\,G\,N}\right\rangle \right]-c^{D/P}\,\left\langle \varsigma_{i}^{A\,F\,F}\,\eta_{k,\mu_{1},\mu_{2}}^{L\,G\,N}\right\rangle +\left(1+c^{D/P}\right)\times \left\langle \Theta \,\left(\varsigma_{i}^{A\,F\,F}-\vartheta_{i}\right)\,\sum_{i^{\prime }\neq i}U_{i,i^{\prime }}\,\eta_{i^{\prime }}^{A\,F\,F}\,\eta_{k,\mu_{1},\mu_{2}}^{L\,G\,N}\right\rangle$$

(13’) $$-c^{D/P}\,\left\langle \sum_{i^{\prime }\neq i}U_{i,i^{\prime }}\,\eta_{i^{\prime }}^{A\,F\,F}\,\eta_{k,\mu_{1},\mu_{2}}^{L\,G\,N}\right\rangle$$

Here we define $\eta_{i}^{A\,F\,F}$ as $\eta_{i}^{A\,F\,F}=\left(\varsigma_{i}^{A\,F\,F}-\vartheta_{i}\right)\cdot \Theta \,\left(\varsigma_{i}^{A\,F\,F}-\vartheta_{i}\right)$. The right-hand sides of the above expressions do not contain $\eta_{i}^{C\,X}$, and we can avoid iterative calculations. The algorithm for the simulations was implemented with these expressions.

## Results

### Experimental Results

#### Orientation Maps in Kittens Reared Under Single-Orientation Exposure

We examined a kitten reared with goggles fitted with zero-power lenses to confirm the effect of lens power on orientation map alteration (Supplementary Figure 3). The transparent zero-power lens did not interfere with the formation of a regular orientation map. This suggests that the marked over-representation of the exposed orientation could not be induced accidentally or spontaneously without non-zero-power lenses. It is natural to believe that the over-representation of the exposed orientation is caused by single-orientation exposure through high-power cylindrical lenses.

**FIGURE 5:**
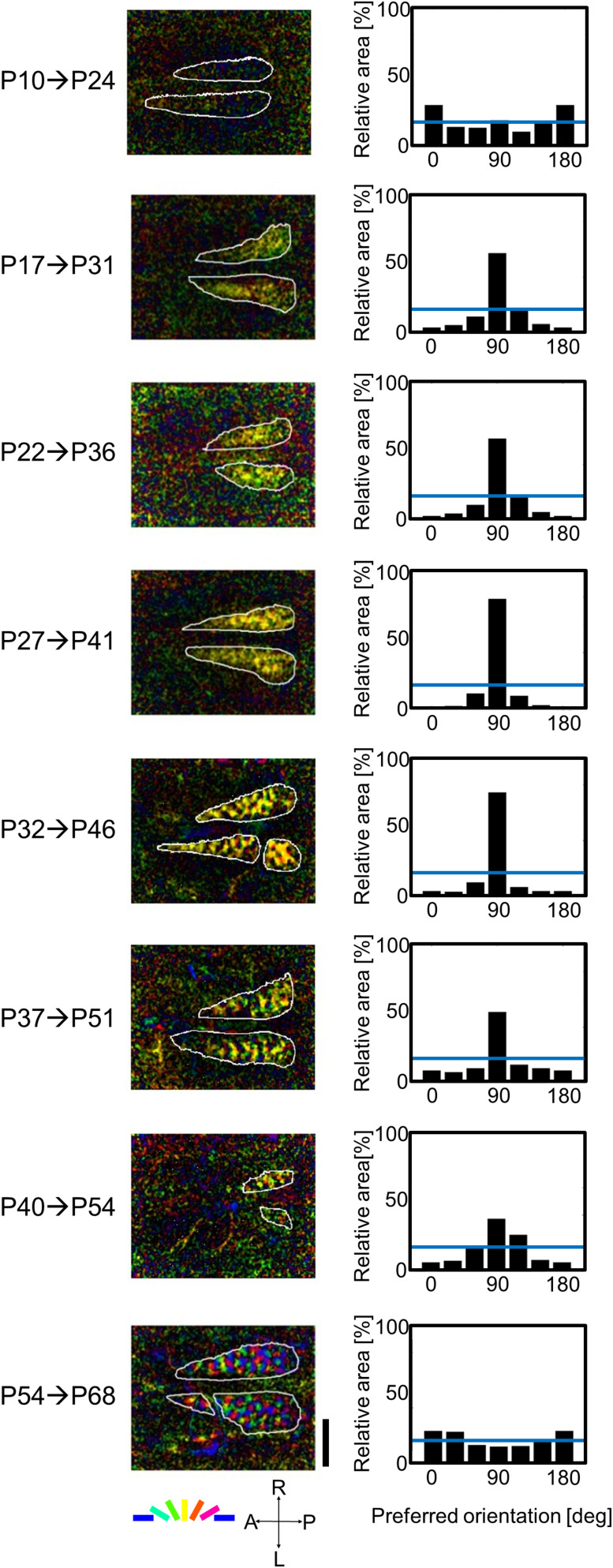
Orientation polar maps and orientation histograms of eight kittens, in which 2-week GR started at P10, P17, P22, P27, P32, P37, P40, and P54, and optical imaging was conducted immediately after GR. The **(left column)** shows orientation polar maps obtained from intrinsic signals evoked by moving grating stimuli of the spatial frequency of 0.5 cpd. Preferred orientation is color coded. The orientation magnitude is indicated by the brightness. The scale bar indicates 4 mm. The **(right columns)** show orientation histograms. The vertical axes of the orientation histograms represent the relative area inside the functionally identified area 17. The horizontal blue lines in the orientation histograms indicate the mean relative area (16.7%). A, anterior; P, posterior; R, right; and L, left in the coordinate system.

Figure 5 shows typical results obtained from the optical imaging of area 17 of eight kittens that experienced only vertical orientation for 2 weeks after they were reared in the normal visual environment without goggles. Ages of GR onset and execution of optical imaging are shown to the left in Figure 5. Orientation polar maps at the ages of P31, P36, P41, P46, P51, and P54 show that cortical domains specifically representing vertical orientation occupied a larger cortical territory (left column in Figure 5). Such over-representation of vertical orientation was clear in orientation histograms (right column in Figure 5). The degree of over-representation differed at different ages of GR onset. The maximal over-representation was seen in the kitten in which 2-week GR started at P27. The over-representation of exposed orientation was not found in the kitten in which 2-week GR started at P54, or rather, horizontal bias and slight under-representation of vertical orientation were observed, as in normally reared young kittens (Supplementary Figure 1). The orientation magnitude (brightness) at the age of P24 is very low, and the shape of the orientation histogram is between a U and a W. If the single-orientation exposure affected orientation selectivity formation from P10 to P24, the histogram should have a single peak at 90°. These observations suggest that the experience-dependent formation of orientation selectivity starts around P24.

#### Sensitive Period Profile for Orientation Plasticity

Here, we quantified orientation plasticity as the normalized relative area of cortical domains devoted to the exposed orientation in area 17 (over-representation index, ORI) at different ages. The definition of the ORI is given as

(23) O⁢R⁢I=Relativearearepresenting 90°[%]-16.667100-16.667

**FIGURE 6:**
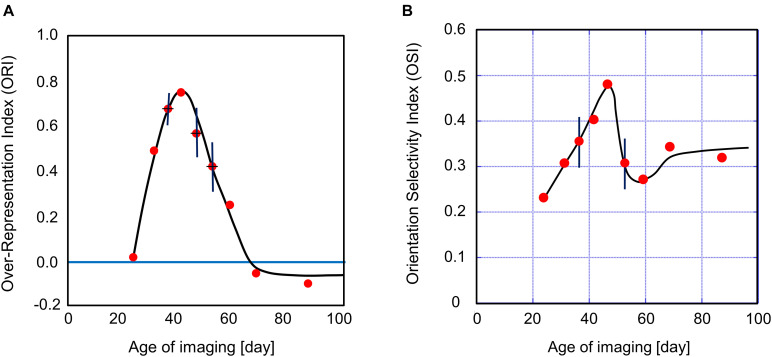
Sensitive period profile for orientation plasticity and changes in the average OSI in area 17 of kittens exposed to single orientation through the goggles for 2 weeks. **(A)** The vertical axis indicates the over-representation index, i.e., the normalized relative area of cortical domains selectively responding to the exposed vertical orientation. The red dots represent the over-representation indices plotted at the age when optical imaging was performed. The most sensitive period is several days around P34. The sensitive period is followed by a period in which single-orientation exposure induces the under-representation of the exposed vertical orientation after about P60 of 2-week GR onset. **(B)** The vertical axis indicates the average OSI over all pixels inside of the functionally identified area 17. The red dots represent the average OSI plotted at the ages when optical imaging was performed. The value of the average OSI was the maximum at P46 and the minimum at P59, which were located in the declining phase of the orientation sensitive period in **(A)**. The error bars shown in **(A,B)** indicate SEs estimated using data in which optical imaging was conducted using more than three cats a few days around the mean age. The cat IDs and numerical data used in **(A,B)** are shown in Supplementary Table 1.

This definition of the ORI is based on 6-bin orientation histograms, as shown in Figure 5. The ORI takes 1 for the complete over-representation of the exposed orientation, and 0 for the unbiased orientation representation. All data points in Figure 6A were taken from kittens reared with vertical goggles for 2 weeks (Supplementary Table 1). This provides the sensitive period profile for orientation plasticity. Although it is difficult to mention exactly when the sensitive period starts and ends owing to the ambiguity of 2 weeks for GR, the profile suggests the existence of such a limited period in early life, in which visual cortical neurons alter their preferred orientations so as to respond strongly to more frequently exposed orientations. According to the profile shown in Figure 6A, it is likely that the sensitive period for orientation plasticity in cat area 17 starts immediately before P24 and ends between P54 and P68. Combined with our experimental observation that orientation maps were not visible in area 17 earlier than P21, the sensitive period for the spatial clustering of neurons with similar orientation preferences may start between P21 and P24, although some neurons are orientation-selective before P20 (Albus and Wolf, 1984).

To examine how the orientation selectivity changes under single-orientation exposure, we define the orientation selectivity index (OSI) of the *i* th pixel inside the region of interest as

(24) $$O\,S\,I\,\left(i\right)=\left(1-\frac{r_{\min}\,\left(i\right)}{r_{\max}\,\left(i\right)}\right)\,\left(1-\frac{w\,\left(i\right)}{180}\right)$$

*r*_*max*_(*i*) and *r*_*min*_(*i*) denote the maximum and minimum stimulus-related intrinsic signals at the *i*th pixel, respectively. *w*(*i*) indicates the full width at half height (FWHH) of the orientation tuning curve of intrinsic signals at the *i* th pixel. In addition, the value of the OSI is determined to be zero when the tuning curve intersects more than three times with the horizontal line at half height, because we cannot determine a preferred orientation uniquely in such a situation. According to Equation (24), OSI increases toward 1 as the orientation tuning curve becomes sharper. The OSI was used as one of indices to characterize orientation selectivity along with orientation magnitude obtained from the vector sum method.

We show the age-dependent changes in the OSI averaged over the imaged area 17 in Figure 6B. The average OSI increased from P24 to P46 of the ages of optical imaging following 2-week GR. Then it decreased until P54, and increased again toward a plateau value. The average OSI under single-orientation exposure is characterized by the dip appearing in the ORI decreasing phase between P46 and P68. This dip may be attributed to the broadening of tuning widths caused by the interference of orientation preferences between the original preferred orientations and the exposed vertical orientation.

#### Orientation Selectivity Modification Under GR Within or Beyond the Sensitive Period

Now, a question is raised about whether the orientation map once markedly over-representing exposed orientation returns to regular orientation maps equally representing all orientations by removing the goggles during the sensitive period. To address this question, we conducted optical imaging three times for the same kitten at P28, P38, and P45 using drifting grating stimuli with the spatial frequency of 0.5 cpd. The first optical imaging was conducted at P28 to observe the intact orientation representation. From P31, the animal was reared with goggles for 1 week, and optical imaging was conducted at P38. After the imaging experiment, the animal was reared without goggles in the normal visual environment for 1 week, and the third optical imaging was performed at P45.

**FIGURE 7:**
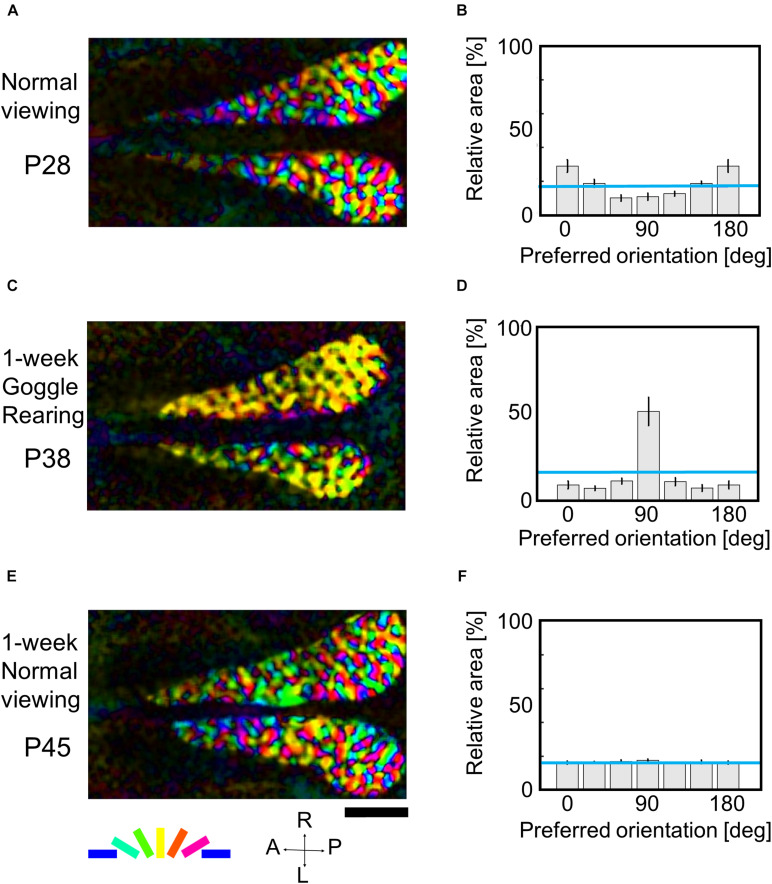
Flexible changes in orientation representation of the same kitten within the sensitive period. **(A,C,E)** Orientation polar maps at P28, P38, and P45, respectively. **(B,D,F)** Orientation histograms at P28, P38, and P45, respectively. The error bars shown in the orientation histograms indicate SEs. All conventions are as in Figure 5. The cat IDs and numerical data used in this figure are shown in Supplementary Table 2.

Figure 7 shows orientation polar maps reconstructed from optical imaging experiments conducted three times on the same kitten (cat ID: GYe). The orientation histograms were obtained from 4 to 6 kittens that underwent the same visual experience and imaging experiments at almost the same age (Supplementary Table 2). We obtained a regular orientation map in functionally identified area 17 (Figure 7A), and the relative areas for preferred orientations were almost uniform (Figure 7B) at P28. One-week GR showed remarkable expansion of cortical domains selectively responding to vertical orientation (Figure 7C). Consequently, the orientation histogram exhibited a skewed distribution biased to the exposed orientation (Figure 7D). One-week normal viewing following 1-week GR, however, altered the orientation map to a normal-like map (Figure 7E), so that the relative areas for preferred orientations changed to a uniform distribution (Figure 7F). The tendency of these changes in the relative areas of iso-orientation domains was reproducibly observed in other kittens goggle-reared in a similar manner (Supplementary Table 2). These findings suggest that even if conspicuous over-representation of a single orientation is induced, a regular orientation map with uniform orientation representation can be restored by keeping kittens in the normal visual environment for 1 week at the latest before P45. In other words, the cat visual cortex has high flexibility to represent any experienced orientation within the sensitive period for orientation plasticity.

**FIGURE 8:**
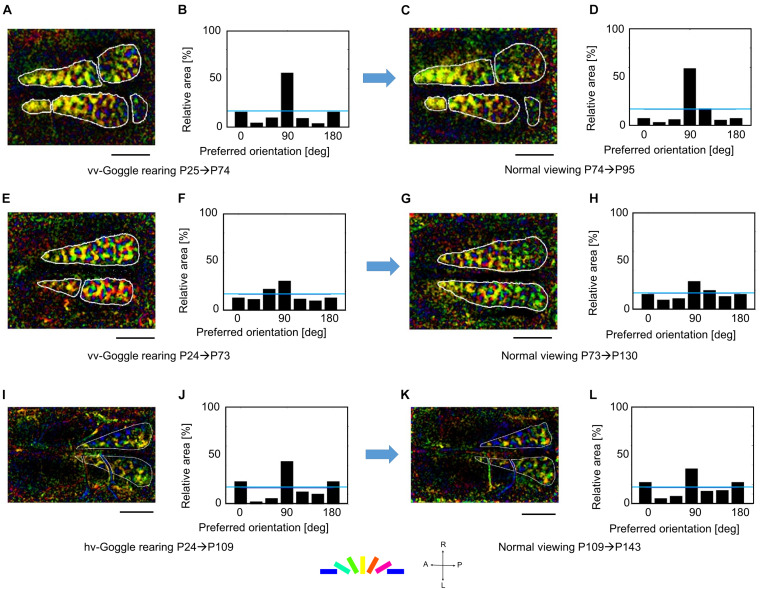
Robustness of orientation representation against visual experience after the end of the sensitive period. **(A,B)** Orientation polar map and histogram at P74 after vertical orientation exposure in the two eyes for 49 days. **(C,D)** Orientation polar map and histogram at P95 after normal viewing for 21 days. **(E,F)** Orientation polar map and histogram at P73 after vertical orientation exposure in the two eyes for 49 days. **(G,H)** Orientation polar map and histogram at P130 after normal viewing for 57 days. **(I,J)** Orientation polar map and histogram at P109 after horizontal and vertical orientation exposure in the left and right eyes, respectively, for 85 days. **(K,L)** Orientation polar map and histogram at P143 after normal viewing for 34 days. All conventions are as in Figure 5. The cat IDs and numerical data in this figure are shown in Supplementary Table 3.

Another question is raised about whether orientation maps exhibiting the marked over-representation of the exposed orientation by prolonged GR beyond the end of the sensitive period can be recovered to regular orientation maps by returning animals to the normal rearing condition. To answer this question, we conducted optical imaging experiments on three kittens that experienced prolonged GR. Figure 8 shows orientation polar maps and histograms of three kittens: two kittens exposed to vertical orientation and one kitten exposed to vertical orientation in the right eye and horizontal orientation in the left eye. The comparison between Figures 8A,C, Figures 8E,G, and Figures 8I,K reveals that orientation polar maps shared common basic structure. Reflecting the preserved over-representation of exposed orientation, pairs of orientation histograms in Figures 8B,D, in Figures 8F,H, and in Figures 8J,L showed remarkable bias toward the exposed orientations, although the relative areas at each preferred orientation were slightly different between the two.

The preservation of orientation representations once-reorganized by prolonged GR should be compared with the finding that only 1-week normal viewing returned the marked over-representation of exposed orientation to the uniform representation between P38 and P45 (Figures 7E,F). These findings suggest that orientation representation was consolidated after P73, until which the time window of orientation plasticity was closed, as shown in Figure 6A.

### Simulation Results

#### Orientation Map Development

**FIGURE 9:**
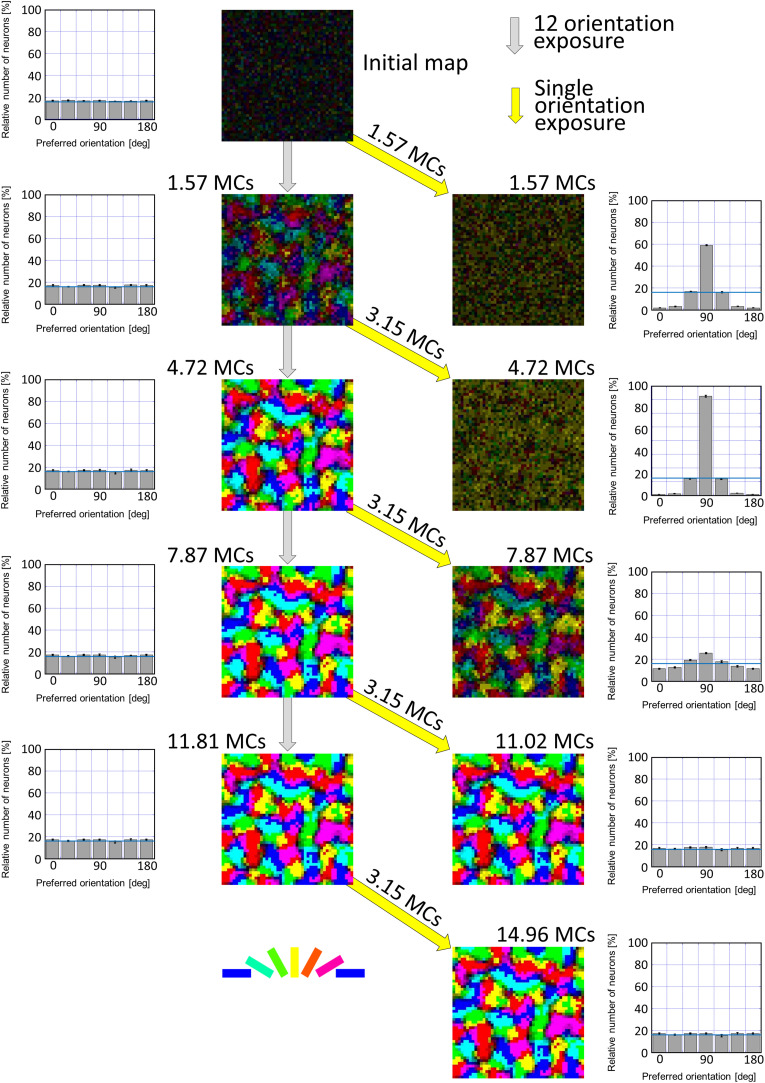
Simulated results of experience-dependent orientation map development. The left half of Figure 9 illustrates the development of an orientation map from an initial map as the simulation step increases. The initial map was produced by random afferent inputs from model LGN neurons to model visual cortical neurons. The simulation for 1.57 MCs formed a regular orientation map, although the segregation into iso-orientation domains was somewhat vague. Once a map structure emerged in an early stage, it was likely that preferred orientations no longer change and only orientation magnitude monotonically increases up to a certain level. The orientation histograms exhibited almost uniform distributions. The simulations under single-orientation exposure were resumed from orientation maps formed under 12-orientation exposure. The right half of Figure 9 shows orientation polar maps and orientation histograms for different durations of the simulations under 12-orientation exposure before the simulation under single-orientation exposure. Different durations of simulations performed under the balanced exposure to 12 orientations induce different degrees of over-representation of exposed orientation. We can see the effect of balanced exposure to 12 orientations in orientation histograms as well as in orientation polar maps. After long simulation under 12-orientation exposure (e.g., 7.87 or 11.81 MC steps), single-orientation exposure for 3.15 MC steps did not alter the orientation map and hence orientation histograms were uniform. This indicates that the sensitive period was closed. The preferred orientation is color-coded shown below. The values of the SE calculated from five trials of simulation with different patterns of initial random afferent inputs are added as vertical bars in the orientation histograms. Numerical data of this figure are shown in Supplementary Table 4 (12-orientation exposure) and Supplementary Table 5 (single-orientation exposure).

First of all, we attempted to reproduce the development of a regular orientation map in the visual cortex of cats reared under normal visual conditions. In computer simulations, model LGN cells were activated by the balanced presentation of grating stimuli drifting in 24 directions (12 orientations). This visual condition is supposed to be a model of the normal visual conditions that animals experience. The left half of Figure 9 illustrates the development of an orientation map as the simulation step increases (from top to bottom in Figure 9). Even a relatively short run of the simulation for 1.57 MC steps formed a regular orientation map, although the segregation into iso-orientation domains was somewhat vague. Once a map structure emerged in an early stage, it was likely that preferred orientations no longer change and only orientation magnitude monotonically increases up to a certain level. The orientation histograms exhibited almost uniform distributions.

The simulation method of synaptic rewiring under single-orientation exposure is the same as the simulation under balanced exposure to 12 orientations except for stimulus patterns presented in the model retina. Model LGN cells were activated by the presentation of only a vertically oriented grating drifting leftward or rightward. This visual condition may correspond to the visual experience of kittens with cylindrical-lens-fitted goggles. We changed the duration of the simulation under 12-orientation exposure before the simulation was performed under single-orientation exposure.

The right half of Figure 9 shows how orientation representations produced under 12-orientation exposure were altered by simulations conducted under single-orientation exposure. Different durations of simulations performed under 12-orientation exposure induce different degrees of over-representation of exposed orientation. We can see the effect of the duration of 12-orientation exposure in orientation histograms as well as in orientation polar maps. After long simulation under 12-orientation exposure (e.g., 7.87 or 11.81 MC steps), single-orientation exposure for 3.15 MC steps did not alter the orientation map and hence orientation histograms were uniform. This indicates that the sensitive period was closed.

#### Sensitive Period Profile for Orientation Plasticity

**FIGURE 10:**
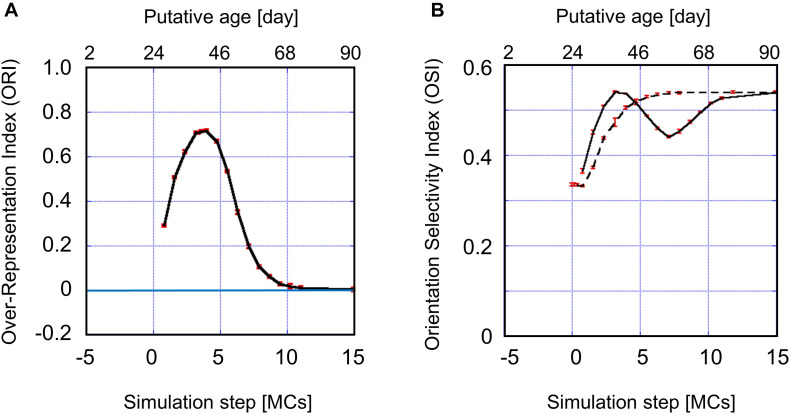
**(A)** Sensitive period profile for orientation plasticity reproduced by computer simulations. The vertical axis indicates the over-representation index (ORI). The horizontal axis indicates the total simulation step *t*, which is given as the sum of durations of 12-orientation exposure and single-orientation exposure. When we assume that 10 days for kittens correspond to 2.25 MCs and map formation starts on P24, we can interpret the simulation step as the age of kittens. Putative ages are shown above the figure. The simulated sensitive period profile behaves similarly to the experimentally observed profile (Figure 6A). The simulated sensitive period is closed around *t*≃10 MCs, corresponding to P68 of the age of cats. **(B)** The average OSI reproduced by computer simulations under 12-orientation exposure alone (dashed curve) and single-orientation exposure following 12-orientation exposure (solid curve). The solid curve is a theoretical counterpart to the experimentally observed OSI behavior in the 2-week goggle-reared kittens (Figure 6B). In the early stage (0 – 5 MCs), the OSI for single-orientation exposure is rapidly increasing and larger than that for 12-orientation exposure. In **(A,B)**, the red vertical bars indicate SEs for five trials of simulation with different patterns of initial random inputs from the LGN to the visual cortex. Numerical data of ORI and OSI are shown in Supplementary Tables 5, 6, respectively.

The values of the ORI obtained from simulations are plotted in Figure 10A. As determined by intrinsic signal optical imaging, clusters of neurons with similar preferred orientations are likely to emerge around P21 to P24, as long as we use the intrinsic signal optical imaging technique. It may therefore be reasonable to set the origin of the simulation time *t=0* to be P24. We may assume that an orientation map is not formed at *t* < 0 because the inverse temperature β is smaller than β_*c*_ (Supplementary Figure 2) at *t* < 0, whereas an orientation map is formed at *t* > 0 in an experience-dependent manner, because β is sufficiently larger than β_*c*_ at *t* > 0. Note that the effective duration of single-orientation exposure should be shorter than 3.15 MCs when it starts before *t* = 3.15 MCs. Consequently, the short-term single-orientation exposure was not sufficient to induce conspicuous over-representation. This may account for the observation that the over-representation of the exposed orientation did not appear clearly in the kitten goggle-reared from P10 to P24 (top row in Figure 5). When we assume that 3.15 MCs corresponds to 2 weeks and the simulation step is linearly dependent on real time, we can reproduce qualitative features of the sensitive period for orientation plasticity. The putative ages are shown at the top of Figure 10. The sensitive period profile for orientation plasticity obtained from simulations agrees well as a whole with the experimentally observed profile. The single-orientation exposure induced under-representation observed later than P68 in Figure 6A was not reproduced in Figure 10A. The dashed curve in Figure 10B indicates the average OSI reproduced by simulations under 12-orientation exposure alone, which predicts the age-dependent OSI changes in normally reared kittens. The solid curve in Figure 10B shows the average OSI reproduced by simulations under single-orientation exposure following 12-orientation exposure, which is a theoretical counterpart to the experimentally observed OSI behavior in the 2-week goggle-reared kittens (Figure 6B). In the early stage (0 – 5 MCs), the OSI for single-orientation exposure more rapidly increases than that for 12-orientation exposure. In the late stage (5 – 10 MCs), the OSI once decreases drastically and then increases again to approach the OSI for 12-orientation exposure around the end of the sensitive period (∼10 MCs). The dip in the solid curve appeared in the decreasing phase (5–10 MCs) of the sensitive period profile shown in Figure 10A.

#### Spatiotemporal Receptive Fields and Orientation Tuning Curves

**FIGURE 11:**
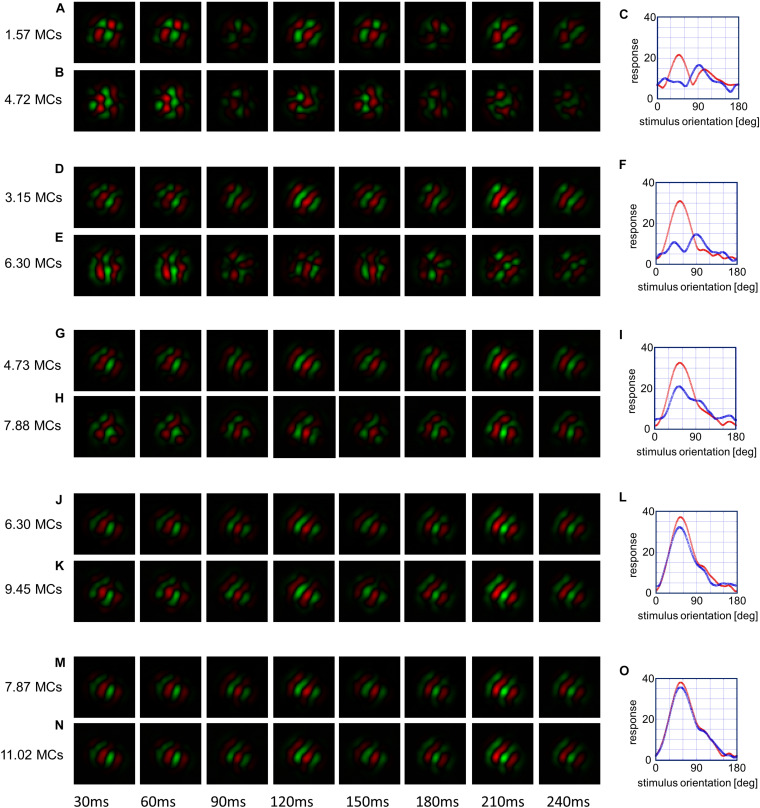
Snapshots of the representative spatiotemporal receptive field of the same neuron. The spatiotemporal receptive field is shown as a spatial receptive field sampled every 10 ms. Changes in snapshots from simulations under 12-orientation exposure for certain MCs to simulation resumed under single-orientation exposure for 3.15 MCs are shown in the pairs of **(A,B)**, **(D,E)**, **(G,H)**, **(J,K)**, and **(M,N)**. Changes in orientation tuning curves are shown in **(C,F,I,L,O)**, where the red and blue curves represent orientation tuning curves immediately before and after single-orientation exposure for 3.15 MCs, respectively.

To obtain a better understanding of the emergence of the dip, we analyzed spatiotemporal receptive fields (DeAngelis et al., 1995) and orientation tuning curves of a typical unidirectional cell in Figure 11. The spatiotemporal receptive field of the *i* th cortical neuron is calculated using the first term of the right-hand side of Equation (14), which represents the afferent component of the membrane potential of the neuron. The receptive field is given by the sum of the products of the number of synaptic inputs and LGN neurons’ activities in response to light and dark spots, as in the reverse correlation method in electrophysiology (DeAngelis et al., 1995). However, we use a simpler estimation method: the LGN spatiotemporal receptive fields $R_{k,\mu_{1},\mu_{2};l}^{L\,G\,N}\,\left(t\right)$ given by Equation (18) are summed up with the weight of the number of synaptic inputs {*n*_*i*,*k*,μ_1_,μ_2__}. That is, $R_{i;l}^{C\,X}\,\left(t\right)=\sum_{k,\mu_{1},\mu_{2}}R_{k,\mu_{1},\mu_{2},l}^{L\,G\,N}\,\left(t\right)\,n_{i,k,\mu_{1},\mu_{2}}$. Figure 11 shows spatiotemporal receptive fields of the same cortical neuron obtained in this way. The red and green regions represent ON and OFF subfields. Since LGN neurons have spatially concentric receptive field, elongated subfields aligned along the axis of the preferred orientation are made of the alignment of four types of LGN neurons that send afferent synaptic inputs to the cortical neurons (Supplementary Figure 4). Figure 11A illustrates the immature spatiotemporal receptive field formed by simulation under the balanced presentation of 12 orientations for1.57 MCs. On the other hand, Figure 11B is the spatiotemporal receptive field formed by simulation under single-orientation exposure for 3.15 MCs, which resumed from simulation under the balanced presentation of 12 orientations for 1.57 MCs. Figure 11C shows the orientation tuning curves for A (red) and B (blue). The maximum response of this neuron to the stimulus orientation of 30° changed to the maximum response to the orientation of 90° by the vertical orientation exposure. The blue tuning curve for H in the left panel shows that a FWHH is about 90° at 7.88 MCs, whereas the red tuning curve for G at 4.73 MCs exhibits a FWHH about 60°. The tuning width for H becomes the broadest among all tuning curves shown in Figure 11. This causes the OSI of this neuron to be small. This implies that orientation tuning of large proportion of neurons at 7.88 MCs tend to be broad and that the average OSI decreases, as observed between 5 and 10 MCs in Figure 10B. The spatiotemporal receptive fields becomes robust after long simulation under the balanced presentation of 12 orientations, and the receptive fields does not change and the tuning curves are almost the same. This indicates that the sensitive period for orientation plasticity of individual neurons ends.

#### Effects of Short-Term GR and Prolonged GR

**FIGURE 12:**
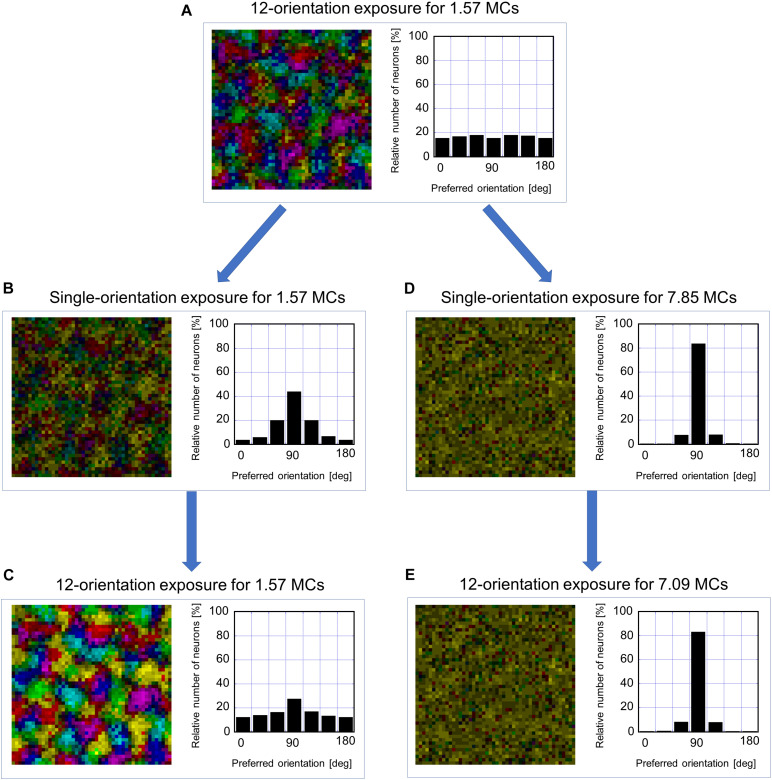
Flexibility or rigidity in orientation representation against the change of visual experience within or after the sensitive period. **(A)** orientation polar map and histogram after simulation under 12-orientation exposure for 1.57 MCs. **(B)** Orientation polar map and histogram after simulation resumed from the state of A under single-orientation exposure for 1.57 MCs. **(C)** Orientation polar map and histogram after simulation resumed from the state of B under 12-orientation exposure for 1.57 MCs. **(D)** Orientation polar map and histogram after simulation resumed from the state of A under single-orientation exposure for 7.85 MCs. **(E)** Orientation polar map and histogram after simulation resumed from the state of D under 12-orientation exposure for 7.09 MCs. The preferred orientation is color-coded as in Figure 9.

To examine the existence of the sensitive period for orientation plasticity, we carried out two simulations: One was simulation under short-term single-orientation exposure and successive recovery, and the other was simulation under prolonged single-orientation exposure and successive long recovery. Figure 12 shows the results of the two simulations. At first, we performed simulation under balanced presentation of 12 orientations for 1.57 MCs (1-week NR in the experiment). The resultant orientation map and orientation histogram are shown in Figure 12A. The two simulations were resumed from the synaptic arrangements after 12-orientation exposure. Short-term single-orientation exposure for 1.57 MCs (1-week GR in the experiment) induced marked over-representation of the exposed orientation (Figure 12B). The successive simulation under 12-orientation exposure for 1.57 MCs restored regular orientation map with clear iso-orientation domains and reduced the over-representation of the vertical orientation (Figure 12C), although the orientation histogram did not return to uniform distribution as shown in Figure 12A. In contrast, prolonged single-orientation exposure for 7.85 MCs (about 5 weeks in the experiment) induced an extreme over-representation (Figure 12D). This over-representation was preserved even after the long-term 12-orientation exposure for 7.09 MCs (4.5 weeks in the experiment) (Figure 12E). The simulation under short-term single-orientation exposure and successive 12-orientation exposure attempted to reproduce the experiment shown in Figure 7. The simulation under prolonged single-orientation exposure and long-term 12-orientation exposure aimed to reproduce the effect of prolonged GR beyond the end of the sensitive period, as shown in Figure 8. Rough features of experimental results were reproduced by the simulations, although the degrees of the over-representation of exposed orientations and to what extent orientation maps return to original maps are different. Based on these simulation results, we may say that in the cat visual cortex, orientation maps are modifiable flexibly by visual experience within the sensitive period, but orientation maps are reorganized by prolonged GR beyond the end of the sensitive period never return to original regular maps. Through the comparisons between the theoretical and experimental studies, it is suggested that the sensitive period for orientation plasticity is closed around P68 (10MCs in the model).

## Discussion

### Onset Age of Visual Experience

According to Albus and Wolf’s electrophysiological experiments (Albus and Wolf, 1984) on kittens, in layer 4, a large proportion of neurons are orientation-selective as early as P14. In contrast, in layers 2/3, orientation-selective neurons were observed first between P14 and P18, and a similar percentage of layer 2/3 neurons remained orientation-selective up to the age of P24. Similar results have been reported by other groups (Wiesel and Hubel, 1974; Blakemore and Van Sluyters, 1975). It is likely that orientation maps in layer 2/3 emerge at certain days between P14 and P24. Although in our group, orientation maps have never been detected in area 17 of kittens younger than P22, Crair et al. (1998) reported that orientation maps existed in area 17 at P14. It is implied that this discrepancy was caused by the possibilities that different camera systems were used for optical imaging and the different spatial frequencies of stimulus gratings might be used. However, they demonstrated that at about P21, orientation maps for the left- and right-eye stimulation become and remain nearly identical to one another in normally reared kittens, whereas maps begin to deteriorate and the two eyes’ maps become less similar in binocularly deprived kittens. These experimental results indicate that visual experience begins to affect orientation maps at P21. On the other hand, based on Figure 6A, single-orientation exposure starts around P24, as mentioned in section “Orientation Map Development.” Taken together, experience-dependent alteration of orientation maps in area 17 of cats may occurs at the age from P21 to P24.

### Lens Power Effects on Orientation Map Alteration

In our experiment, 2-week GR of kittens exposed to vertical orientation with plano-convex lenses of +67 diopter from P27 exhibited the maximum over-representation of the exposed orientation. When GR started at P27 or earlier, pinwheel centers around which all orientations are arranged continuously were hard to identify (Figure 5) because all orientations were not represented. Sengpiel et al. (1999) also observed the over-representation of the exposed orientation. However, the cortical territory representing the exposed orientation was moderately expanded, but they emphasized that basic structure of orientation maps was robust and all orientations were represented. Such a difference may be attributed partly to the lens power (−25 diopter vs. +67 diopter) and partly to the time for single-orientation exposure a day (6 h vs. 24 h). The images transformed through lenses of these powers can be seen in Figure 1 of Tanaka et al. (2006). In a recent experiment on kittens reared with discordant goggles in the two eyes, plano-concave lenses of −10 diopter were adopted, which were fitted to a rubber mask (Cloherty et al., 2016). Such lenses of weak power showed that orientation maps, in which all preferred orientation were represented, enabled to analyze geometric properties of pinwheel centers. In this sense, visual restriction with week-power lenses would be advantageous to elucidate the effects of visual experience on the geometric relationships among orientation gradients, pinwheel centers and ocular dominance maps, by comparing with simulation results (Carreira-Perpinãń et al., 2005; Giacomantonio et al., 2010; Cloherty et al., 2016).

### Comparisons Among Models

Some abstract models such as the self-organizing map (SOM) (Obermayer et al., 1990; Kohonen, 1997) and the elastic net model (Durbin and Mitchison, 1990; Carreira-Perpinãń et al., 2005; Goodhill, 2007) may provide a computational principle of map formation optimizing the quantities such as “coverage” and “continuity”. In these models, based on the *a priori* assumption that each neuron has receptive field parameters such as preferred orientation, preferred direction of motion, and ocular dominance, these parameters are represented over the visual cortical surface as continuously as possible. Actually, regular and bandpass-filter-like orientation maps with pinwheel centers have been well reproduced, as observed in cats, ferrets, and macaques. However, experimental results were not always consistent with the continuity assumption of visual features. In electrophysiological recording in the visual cortex of cats reared with stripe environment, the preferred orientation did not change continuously along the electrode track due to the singular intervention of orientation non-selective or unresponsive neurons (Figure 5 in Stryker et al., 1978). Later we will demonstrate that our model reproduces the phenomenon of discontinuous orientation representations in the cats reared in the stripe environment.

On the other hand, another type of models has been proposed to reproduce orientation maps on the basis of the Hebbian learning (Hebb, 1949) of afferent synaptic inputs from the LGN (Miller, 1992, 1994; Miyashita and Tanaka, 1992; Miyashita et al., 1997). These models describe the activity-dependent self-organization of synaptic inputs explicitly, called correlation-based learning model. Unfortunately, these models generated low-pass filter-like orientation maps with large fluctuation in the size of iso-orientation domains (Erwin et al., 1995). This may be related to the absence of the *a priori* coverage assumption. In addition, this type of models requires some normalization constraints on the synaptic strength (Goodhill and Barrow, 1994; Miller, 1994; Miller and Mackay, 1994) or the number of synaptic inputs (Tanaka and Miyashita, 2009) to prevent visual cortical neurons from receiving exuberant synaptic inputs from a particular LGN neuron. If the correlation-based learning models are improved getting rid of these defects, they may be able to give us a logical linkage between molecular mechanisms for synaptic rewiring to the experience-dependent development of orientation maps. Thus, we attempted to build a synaptic rewiring model, refining the correlation-based learning so that it can reproduce band-pass filter-like orientation maps.

For microscopic mechanisms of synaptic rewiring, we focused on BDNF and its related receptors, reducing numerous molecules that may be related to synaptic rewiring, and suggest that activity-dependent and independent release of BDNF is an essential for experience-dependent orientation map formation/reorganization. For intracellular Ca^2+^ concentration, which controls the release of BDNF and tPA from the postsynaptic site, we simply assumed the enhancement of Ca^2+^ concentration by the coincidental bAP (Nevian and Sakmann, 2004). The model obtained on this basis resolved the defects of the correlation-based learning, and would be a minimal model to show the sensitive period for orientation plasticity as well as biologically plausible orientation maps. In addition, the model accounted for the formation of spatiotemporal receptive fields of simple cells based on self-organized afferent inputs from four types of LGN neurons. This reproducibility is another successful demonstration of the model at the electrophysiological level.

The Hebbian coincidence of presynaptic activity with the postsynaptic activity (Hebb, 1949) has been assumed, in most mathematical models for activity-dependent synaptic plasticity, and the coincidence level is reflected by Ca^2+^ influx through the NMDA channel (e.g., Graupner and Brunel, 2010). In our model, we assumed the enhancement of intracellular Ca^2+^ concentration associated with the arrival of bAPs, in addition to Ca^2+^ influx through NMDA channels triggered by the coincidence of presynaptic activity with postsynaptic membrane depolarization (Nevian and Sakmann, 2004). This triple coincidence causes a large increase in Ca^2+^ concentration in the vicinity of the synaptic contact on the postsynaptic dendritic spine, and release the sufficient amount of mBDNF locally at the synaptic contact, which works to be advantageous for the synaptic contact in the competition with other synaptic contacts. If this triple coincidence happens at a newly formed synapse, the synapse may be stabilized to survive longer. On the other hand, the simple coincidence of the presynaptic activity with the postsynaptic depolarization induces a moderate increase in Ca^2+^ concentration, which facilitates to release locally the larger amount of proBDNF relatively to that of mBDNF. This tends to act to be advantageous for the other synaptic contacts on the same spine, and likely to eliminate the synaptic contact in question. This coincidence may serve to normalize the number of synaptic connections to avoid a situation in which exuberant afferent inputs from a presynaptic neuron occupy a large portion of dendritic sites of a postsynaptic neuron. Such a constraint is indispensable to generate simple cell-like receptive fields (Miyashita and Tanaka, 1992; Miyashita et al., 1997). Otherwise, receptive fields of visual cortical neurons become a center-surround antagonistic receptive fields, as seen in the LGN relay neurons.

Although the correlation-based learning models reproduced simple-cell receptive fields characterized by orientation and phase parameters, and generated orientation map-like patterns. However, the orientation maps were low-pass filter-like, as indicates that the size of iso-orientation domains was highly fluctuating, as Erwin et al. (1995) pointed out. The emergence of low-pass filter-like maps is speculated to be caused by the mutual interference between the preferred orientation map and the receptive field phase map, because this type of models tends to regularly arrange any receptive field parameter over the model cortex. It should be noted that the receptive field phase seems to be randomly distributed tangentially along the cat visual cortex (DeAngelis et al., 1999). Simulations of the present model with the triple coincidence demonstrated that receptive field phases are randomly arranged over the model cortex (data not shown). Driving out the phase maps results in the formation of regular orientation maps characterized by a band-pass filter-like structure. This property of orientation maps reproduced by the present model can be confirmed by the power spectra shown in Supplementary Figure 2.

If the postsynaptic neuron’s firing is blocked in some way, what happens? According to the present model, the activity-dependent release of proBDNF is dominant over that of mBDNF, when the absence of bAP does not enhance Ca^2+^ concentration sufficiently. In Equation (13), the first term on the right-hand side vanishes due to the suppression of bAP, and only the second term remains. In this situation, the activity-dependent component of the synapse growth rate becomes negative. This indicates that the synaptic rewiring probability that a weaker synaptic contact survives and a stronger contact is retracted becomes larger than the probability of the opposite process. Reiter and Stryker (1988) have reported the reversal shift of ocular dominance to the deprived eye but not to the non-deprived eye in monocularly deprived kittens whose visual cortices were infused with the GABA receptor agonist muscimol. This odd phenomenon should be reproduced because the synapse growth rate always takes more negative values for non-deprived-eye inputs than for deprived-eye inputs, although the present model was not extended to the model with binocular inputs. This experimental finding is expected to support any models based on the assumption that LTP is induced for high intracellular Ca^2+^ concentration and LTD is induced for moderate Ca^2+^ concentration by manipulating the threshold for the switching between LTP induction and LTD induction.

As mentioned in section “Hypothetical Mechanisms of Synaptic Rewiring,” our hypothetical mechanism is basically the same as the BCM theory, although the explicit variable representing the Ca^2+^ concentration does not appear in our model. However, one of the differences between the BCM model and ours rather consists in how to use the rule for synaptic update. The synapse growth rate corresponds to the synaptic efficacy change in the BCM model. In our model, only a synaptic contact having the maximum synapse growth rate can be stabilized to survive, and the other contacts are eliminated. Even if the synapse growth rate of a synaptic contact is positive, it will be defeated by another contact with larger synapse growth rate. This process is realized by the algorithm in which a synaptic contact is replaced with a stronger contact, keeping the total number of synaptic contacts on a cortical neuron constant. However, after synaptic rewiring is completed (Figure 3E) and only a single axonal bouton contacts with the spine in question, the sign of the synapse growth rate determines the direction of the synaptic efficacy toward LTP (positive) or LTD (negative).

### Tuning Curves Changed by Single-Orientation Exposure

In Figure 6B, the average OSI seen experimentally changed depending on age in a non-monotonic manner. Figure 10B obtained from simulations also showed a similar behavior of the average OSI. At the early stage of the sensitive period, the orientation tuning curves of most neurons are immature and broad. Restricted exposure to vertical orientation for 2 weeks (3.15 MCs in the model) alters their preferred orientations toward the exposed orientation. In Figure 10B, the OSI for single-orientation exposure was larger than that for 12-orientation exposure from 0 to 5 MCs. This suggests that orientation selectivity is sharper than that of kittens reared in the normal visual environment at this stage. At the late stage of the sensitive period, the orientation selectivity is almost matured, and the orientation tuning curves generated by normal visual experience (12-orientations in the model) tend to be restored despite single-orientation exposure. Both in Figures 6B, 10B, the dip of the OSI for single-orientation exposure appeared at the ORI declining phase in the sensitive period. At this stage, the OSI of individual neurons are likely to decrease as the orientation tuning curves become broadened or bimodal. In the bimodal orientation tuning curves, one peak appears at the original preferred orientation and the other at the exposed orientation. Such modifications of tuning curves are caused by the disruption of ON or OFF responsive subfields elongated in parallel to the preferred orientation under the influence of the exposed orientation. This can be seen in the comparisons between the changes in responsive subfields (Figure 11D,E,G,H) and the changes in tuning curves (the red curves to blue curves in Figures 11F,I). Broad tuning curves after single-orientation exposure (e.g., blue curve in Figure 11I) are likely to appear in the transition from the bimodal distribution (e.g., blue curve in Figure 11F) to the unimodal distribution (e.g., blue curve in Figure 11L).

**FIGURE 13:**
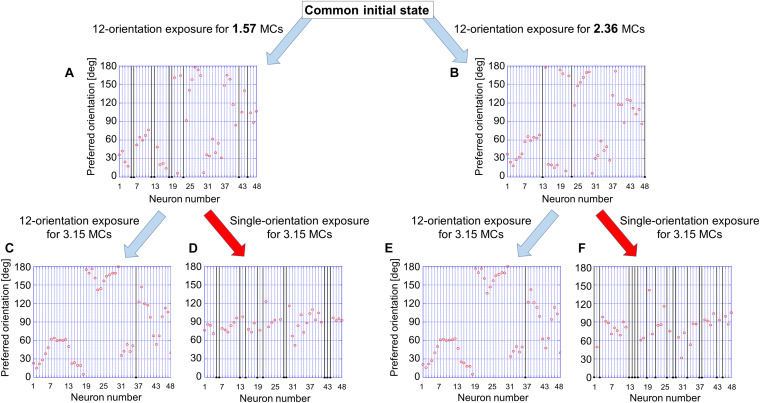
Preferred orientation as a function of the neuron’s position along the same line in the model visual cortex for different stimulation conditions and different simulation steps. **(A,B)** The preferred orientations changing with the one-dimensional position, and positions of orientation-nonselective neurons obtained from simulations under 12-orientation exposure for 1.57 MCs and 2.36 MCs, respectively. A pattern of initial random inputs from the model LGN to the model cortex was common for **(A,B)**. **(C,D)** The preferred orientations and orientation-nonselective neurons along the same line obtained from simulations resumed for 3.15 MCs from **(A)** under 12-orientation and single-orientation exposures, respectively. **(E,F)** The preferred orientations and orientation-nonselective neurons along the same line obtained from simulations resumed for 3.15 MCs from **(B)** under 12-orientation and single-orientation exposures, respectively. Preferred orientations are indicated by red circles, and positions of orientation-nonselective neurons are indicated by black dots with vertical bars. Numerical data are shown in Supplementary Table 7.

Related to this, in orientation maps obtained from simulations under single-orientation exposure (e.g., map at 4.73 MCs in Figure 9), almost continuous changes in preferred orientations of neurons (bright pixels) were interleaved with orientation-nonselective neurons (dark pixels), whose orientation tuning curves were not unimodal. The emergence of non-selective neurons between clusters of orientation-selective neurons can be more clearly observed in Figure 13. Starting from a common initial state which receives random afferent inputs from the model LGN, simulations were conducted under 12-orientation exposure for 1.57 MCs or 2.36 MCs at the beginning, and then resumed under the same condition or single-orientation exposure for 3.15 MCs. Figure 13 shows typical one-dimensional changes in the preferred orientation sampled from neurons along the same line on the model cortex (red circles). It also shows the positions of orientation-nonselective neurons along the same line (black dots with vertical lines), where orientation-nonselective neurons were defined by neurons with OSI = 0 and orientation magnitudes (in the sense of the vector sum method) smaller than a half of the magnitude averaged over the model cortex. Orientation-nonselective neurons were shown to appear interleaving clusters of orientation-selective neurons even in the streams of simulations under 12-orientation exposure as in A–C, or B–E. There was a tendency that the number of orientation-nonselective neurons decreases as the simulation step of 12-orientation exposure. In contrast, after 3.15 MCs of simulations under single-orientation exposure following 12-orientation exposure, many nonselective neurons appeared disrupting the gradual changes in the preferred orientation, as shown in Figures 13D,F. These simulation results are consistent with the experimental findings reported by Stryker et al. (1978). They reported that the regions of orientation-selective cells were separated by gaps containing only non-selective or unresponsive cells along the electrode track in the visual cortex of cats reared viewing horizontal stripes with both eyes (see Figure 5C in Stryker et al., 1978).

### What Determines the Closure of the Sensitive Period?

Figures 7, 8 indicate the existence of a period in which the orientation representation is modifiable by altered visual experience, and orientation maps are unable to change after the period. The factor determining the end of the sensitive period consists in the probability of postsynaptic dendritic spines change their morphology to reach varicosities along the geniculo-cortical axons to make transient synaptic contacts. We assumed that the expression of p75^NTR^ changes with age in early life, and the binding of proBDNF secreted from the constitutive pathway to p75^NTR^ facilitates the elimination of labile synaptic contacts. This elimination is considered being caused by the decrease of the probability that a postsynaptic spine moves and changes its shape to encounter axonal varicosities to make a transient synaptic contact, as expressed by Equation (11). We assumed the age-dependent increase in the expression of p75^NTR^ as seen in Equation (12). As a result, the frequency of a postsynaptic spine making another transient synaptic contact for rewiring decreases, and the orientation map modifiability disappears. Consequently, the end of the sensitive period is determined by the time when the probability given by Equation (11) becomes sufficiently small. So far, the regulatory pathway of BDNF secretion has attracted much attention, because the pathway is considered being relevant to activity-dependent synaptic plasticity underlying the elaboration of neural networks during development, and in adult learning and memory. However, the activity-independent mechanism of BDNF secretion works in the constitutive pathway in addition to the activity-dependent mechanism in the regulatory pathway. In the present model, activity-independent mechanisms of BDNF secretion and p75^NTR^ expression, which are related to the inverse temperature β and the closure of the sensitive period, respectively, are important factors to obtain a better understanding of how synaptic rewiring occurs and how visual experience influences orientation map development.

It has been widely accepted that proBDNF and mBDNF have opposing functions in the early development of neuromuscular junctions, which induces competition among different inputs to eliminate inadequate inputs and stabilize the most adequate input as a winner (Je et al., 2012, 2013). Analogously, we hypothesized that mBDNF and proBDNF in the visual cortex during postnatal development serve to stabilize synaptic contacts that transmit neural signals most efficiently and eliminate other inefficient contacts. The survival or extinction of transient synaptic contacts is determined by the difference of the synapse growth rates in the vicinity of individual contacts [Figures 3B–D, Equation (7)]. Since the amounts of secretion of mBDNF and proBDNF depend on the intracellular Ca^2+^ concentration, one would be able to argue such synaptic rewiring in terms of the dynamics of Ca^2+^ concentration alone without referring to the roles of BDNF, neurotrophin receptors, and tPA. However, the present model built explicitly on those molecules is more likely to account for the sensitive period for synaptic plasticity than other models based only on Ca^2+^ dynamics. Particularly, the age-dependent expression of p75^NTR^ considered in the present model is determinant. There are findings that the mRNA level of p75^NTR^ decreases in the visual cortex of rats around the sensitive period (Bracken and Turrigiano, 2009) and proBDNF collapses neurite outgrowth through p75^NTR^ activation (Sun et al., 2012). In our model, assuming that the p75^NTR^ expression steeply decreased around eye opening increases around the end of the sensitive period, which reduces the chance of participation of candidate synaptic contacts to synaptic competition owing to the pruning of dendritic spines and/or terminaux boutons along the presynaptic axons, the model successfully reproduced the sensitive period profile (Figure 10A compared with Figure 6A). Although the present model remains to be extended to a model with the left and right eye inputs, it may be able to account for previous experimental results: The intracortical blockade of tPA or plasmin selectively prevents recovery from monocular-deprivation induced dysfunction during reverse occlusion (Müller and Griesinger, 1998); and the cortical infusion of exogenous BDNF inhibits ocular dominance column formation during postnatal development (Cabelli et al., 1995). Furthermore, the model may contribute to the designing of experiments to explore possible methods for the restoration of developmental plasticity even in the adulthood.

## Data Availability Statement

The raw data supporting the conclusions of this article will be made available by the authors, without undue reservation, to any qualified researcher.

## Ethics Statement

The animal study was reviewed and approved by the Institutional Animal Research Committee at RIKEN and performed in accordance with the guideline of the Japanese Physiological Society.

## Author Contributions

ST built the mathematical model of synaptic rewiring and wrote the manuscript. MM coded the Fortran programs based on the synaptic rewiring algorithm and carried out the simulations. NW carried out the simulations and provided the simulation results. KO’H and TT provided the part of the experimental data. JR built a systematic analysis method of evoked intrinsic signals. All the authors contributed to the article and approved the submitted version.

## Conflict of Interest

NW was employed by the company All Nippon Airways Co., Ltd. The remaining authors declare that the research was conducted in the absence of any commercial or financial relationships that could be construed as a potential conflict of interest.

## References

[B1] Albus K. Wolf W. (1984). Early post-natal development of neuronal function in the kitten’s visual cortex: a laminar analysis. *J. Physiol.* 348 153–185. 10.1113/jphysiol.1984.sp015104 6716282 PMC1199396

[B2] Araque A. Parpura V. Sanzgiri R. P. Haydon P. G. (1999). Tripartite synapses: glia, the unacknowledged partner. *Trends Neurosci.* 22 208–215. 10.1016/s0166-2236(98)01349-6 10322493

[B3] Baho E. Chattopadhyaya B. Lavertu-Jolin M. Mazziotti R. Awad P. N. Chehrazi P. (2019). p75 neurotrophin receptor regulates the timing of the maturation of cortical parvalbumin cell connectivity and promotes ocular dominance plasticity in adult visual cortex. *J. Neurosci.* 39 4489–4510. 10.1523/jneurosci.2881-18.2019 30936240 PMC6554620

[B4] Bear M. F. Rittenhouse C. D. (1999). Molecular basis for induction of ocular dominance plasticity. *J. Neurobiol.* 41 83–91. 10.1002/(sici)1097-4695(199910)41:1<83::aid-neu11>3.0.co;2-z 10504195

[B5] Berardi N. Pizzorusso T. (2004). Extracellular matrix and visual cortical plasticity: freeing the synapse. *Neuron* 44 905–908. 10.1016/s0896-6273(04)00796-2 15603733

[B6] Bienenstock E. L. Cooper L. Munro P. (1982). Theory for the development of neuron selectivity: orientation specificity and binocular interaction in visual cortex. *J. Neurosci.* 2 32–48. 10.1523/jneurosci.02-01-00032.1982 7054394 PMC6564292

[B7] Binder D. K. Scharfman H. E. (2004). Brain-derived neurotrophic factor. *Growth Fact.* 22 123–131. 10.1080/08977190410001723308 PMC2504526 15518235

[B8] Blakemore C. Cooper G. F. (1970). Development of the brain depends on visual environment. *Nature* 228 477–478. 10.1038/228477a0 5482506

[B9] Blakemore C. Van Sluyters R. C. (1975). Innate and environmental factors in the development of the kitten’s visual cortex. *J. Physiol.* 248 663–716. 10.1113/jphysiol.1975.sp010995 1151843 PMC1309546

[B10] Bonhoeffer T. Grinvald A. (1991). Iso-orientation domains in cat visual cortex are arranged in pinwheel-like patterns. *Nature* 353 429–431. 10.1038/353429a0 1896085

[B11] Bonhoeffer T. Grinvald A. (1996). “Optical imaging based on intrinsic signals: the methodology,” in *Brain Mapping: The Methods*, eds Toga A. Mazziotta J. C., (San Diego: Academic Press), 55–97.

[B12] Bracken B. K. Turrigiano G. G. (2009). Experience-dependent regulation of TrkB isoforms in rodent visual cortex. *Dev. Neurobiol.* 69 267–278. 10.1002/dneu.20701 19224567 PMC2834411

[B13] Cabelli R. J. Hohn A. Shatz C. J. (1995). Inhibition of ocular dominance column formation by infusion of NT-4/5 or BDNF. *Science* 267 1662–1666. 10.1126/science.7886458 7886458

[B14] Carreira-Perpinãń M. Á Lister R. Goodhill G. J. (2005). A computational model for the development of multiple maps in primary visual cortex. *Cereb. Cortex* 15 1222–1233. 10.1093/cercor/bhi004 15616135

[B15] Cloherty S. L. Hughes N. J. Hietanen M. A. Bhagavatula P. S. Goodhill G. J. Ibbotson M. R. (2016). Sensory experience modifies feature map relationships in visual cortex. *eLife* 5:e13911. 10.7554/eLife.13911 27310531 PMC4911216

[B16] Crair M. C. Gillespie D. C. Stryker M. P. (1998). The role of visual experience in the development of columns in cat visual cortex. *Science* 279 566–570. 10.1126/science.279.5350.566 9438851 PMC2453000

[B17] Cunha C. Brambilla R. Thomas K. L. (2010). A simple role for BDNF in learning and memory? *Front. Mol. Neurosci.* 3:1. 10.3389/neuro.02.001.2010 20162032 PMC2821174

[B18] DeAngelis G. C. Ghose G. M. Ohzawa I. Freeman R. D. (1999). Functional micro-organization of primary visual cortex: receptive field analysis of nearby neurons. *J. Neurosci.* 19, 4046–4064. 10.1523/JNEUROSCI.19-10-04046.1999 10234033 PMC6782727

[B19] DeAngelis G. C. Ohzawa I. Freeman R. D. (1995). Receptive-field dynamics in the central visual pathways. *Trends Neurosci.* 18 451–458. 10.1016/0166-2236(95)94496-r 8545912

[B20] Durbin R. Mitchison G. (1990). A dimension reduction framework for understanding cortical maps. *Nature* 343 644–647. 10.1038/343644a0 2304536

[B21] Erwin E. Obermayer K. Schulten K. (1995). Models of orientation and ocular dominance columns in the visual cortex: a critical comparison. *Neural Comput.* 7 425–468. 10.1162/neco.1995.7.3.425 8935959

[B22] Fagiolini M. Hensch T. K. (2000). Inhibitory threshold for critical-period activation in primary visual cortex. *Nature* 404 183–186. 10.1038/35004582 10724170

[B23] Fagiolini M. Pizzorusso T. Berardi N. Domenici L. Maffei L. (1994). Functional postnatal development of the rat primary visual cortex and the role of visual experience: dark rearing and monocular deprivation. *Vis. Res.* 34 709–720. 10.1016/0042-6989(94)90210-0 8160387

[B24] Frey U. Frey S. Schollmeier F. Krug M. (1996). Influence of actinomycin D, a RNA synthesis inhibitor, on long-term potentiation in rat hippocampal neurons in vivo and in vitro. *J. Physiol.* 490 703–711. 10.1113/jphysiol.1996.sp021179 8683469 PMC1158708

[B25] Fukai T. Tanaka S. (1997). A simple neural network exhibiting selective activation of neuronal ensembles: from winner-take-all to winner-share-all. *Neural Comput.* 9 77–97. 10.1162/neco.1997.9.1.77 9117902

[B26] Giacomantonio C. E. Ibbotson M. R. Goodhill G. J. (2010). The influence of restricted orientation rearing on map structure in primary visual cortex. *Neuroimage* 52 875–883. 10.1016/j.neuroimage.2009.12.066 20035888

[B27] Gilbert C. D. Das A. Ito M. Kapadia M. Westheimer G. (1996). Spatial integration and cortical dynamics. *Proc. Natl. Acad. Sci. U.S.A.* 93 615–622. 10.1073/pnas.93.2.615 8570604 PMC40100

[B28] Goodhill G. J. (2007). Contributions of theoretical modeling to the understanding of neural map development. *Neuron* 56 301–311. 10.1016/j.neuron.2007.09.027 17964247

[B29] Goodhill G. J. Barrow H. G. (1994). The role of weight normalization in competitive learning. *Neural Comput.* 6:1994.

[B30] Graupner M. Brunel N. (2010). Mechanisms of induction and maintenance of spike-timing dependent plasticity in biophysical synapse models. *Front. Comput. Neurosci.* 4:136. 10.3389/fncom.2010.00136 20948584 PMC2953414

[B31] Greenberg M. E. Xu B. Lu B. Hempstead B. (2009). New insights in the biology of BDNF synthesis and release: implications in CNS function. *J. Neurosci.* 29 12764–12767. 10.1523/jneurosci.3566-09.2009 19828787 PMC3091387

[B32] Hebb D. O. (1949). *The Organization of Behavior.* New York, NY: Wiley.

[B33] Hirsch H. V. B. Spinelli D. N. (1970). Visual experience modifies distribution of horizontally and vertically oriented receptive fields in cats. *Science* 168 869–871. 10.1126/science.168.3933.869 5444065

[B34] Hofbauer J. Sigmund K. (1988). *The Theory of Evolution and Dynamical System, London Mathematical Society Student Texts.* New York, NY: Cambridge University Press.

[B35] Hubel D. H. Wiesel T. N. (1962). Receptive fields, binocular interaction and functional architecture in the cat’s visual cortex. *J. Physiol.* 160 106–154. 10.1113/jphysiol.1962.sp006837 14449617 PMC1359523

[B36] Hubel D. H. Wiesel T. N. (1965). Comparison of the effects of unilateral and bilateral eye closure on cortical unit responses in kittens. *J. Neurophysiol.* 28 1029–1040. 10.1152/jn.1965.28.6.1029 5883730

[B37] Hubel D. H. Wiesel T. N. (1968). Receptive fields and functional architecture of monkey striate cortex. *J. Physiol.* 195 215–243. 10.1113/jphysiol.1968.sp008455 4966457 PMC1557912

[B38] Hubel D. H. Wiesel T. N. (1974). Sequence regularity and geometry of orientation columns in the monkey striate cortex. *J. Comp. Neurol.* 158 267–294. 4436456 10.1002/cne.901580304

[B39] Hubel D. H. Wiesel T. N. (1977). Ferrier lecture. Functional architecture of macaque monkey visual cortex. *Proc. R. Soc. Lond Ser. B* 198 1–59. 10.1098/rspb.1977.0085 20635

[B40] Hubel D. H. Wiesel T. N. LeVay S. (1977). Plasticity of ocular dominance columns in monkey striate cortex. *Philos. Trans. R. Soc. B* 278 131–163. 10.1098/rstb.1977.0050 19791

[B41] Je H. S. Yang F. Ji Y. Nagappan G. Hempstead B. L. Lu B. (2012). Role of pro-brain-derived neurotrophic factor (proBDNF) to mature BDNF conversion in activity-dependent competition at developing neuromuscular synapses. *Proc. Natl. Acad. Sci. U.S.A.* 109 15924–15929. 10.1073/pnas.1207767109 23019376 PMC3465384

[B42] Je H. S. Yang F. Ji Y. Potluri S. Fu X.-Q. Luo Z.-G. (2013). ProBDNF and mature BDNF as punishment and reward signals for synapse elimination at mouse neuromuscular junctions. *J. Neurosci.* 33 9957–9962. 10.1523/jneurosci.0163-13.2013 23761891 PMC3682390

[B43] Kim D.-S. Matsuda Y. Ohki K. Ajima A. Tanaka S. (1999). Geometrical and topological relationships between multiple functional maps in cat primary visual cortex. *Neuroreport* 10 2515–2522. 10.1097/00001756-199908200-00015 10574362

[B44] Koester H. J. Sakmann B. (1998). Calcium dynamics in single spines during coincident presynaptic and postsynaptic activity depend on relative timing of back-propagating action potentials and subthreshold excitatory postsynaptic potentials. *Proc. Natl. Acad. Sci. U.S.A.* 95 9596–9601. 10.1073/pnas.95.16.9596 9689126 PMC21384

[B45] Koester H. J. Sakmann B. (2000). Calcium dynamics associated with action potentials in single nerve terminals of pyramidal cells in layer 2/3 of the young rat neocortex. *J. Physiol.* 529 625–646. 10.1111/j.1469-7793.2000.00625.x 11118494 PMC2270226

[B46] Kohonen T. (1997). *Self-Organizing Maps.* Berlin: Springer-Verlag.

[B47] Kuczewski N. Porcher C. Lessmann V. Medina I. Gaiarsa J. L. (2009). Activity-dependent dendritic release of BDNF and biological consequences. *Mol. Neurobiol.* 39 37–49. 10.1007/s12035-009-8050-7 19156544 PMC5352827

[B48] Lesmann V. Brigadski T. (2009). Mechanisms, locations, and kinetics of synaptic BDNF secretion: an update. *Neurosci. Res.* 65 11–22. 10.1016/j.neures.2009.06.004 19523993

[B49] LeVay S. Wiesel T. N. Hubel D. H. (1980). The development of ocular dominance columns in normal and visually deprived monkeys. *J. Comp. Neurol.* 191 1–51. 10.1002/cne.901910102 6772696

[B50] Markram H. Lubke J. Frotscher M. Sakmann B. (1997). Regulation of synaptic efficacy by coincidence of postsynaptic APs and EPSPs. *Science* 275 213–215. 10.1126/science.275.5297.213 8985014

[B51] Mataga N. Mizuguchi Y. Hensch T. K. (2004). Experience-dependent pruning of dendritic spines in visual cortex by tissue plasminogen activator. *Neuron* 44 1031–1041. 10.1016/j.neuron.2004.11.028 15603745

[B52] Mataga N. Nagai N. Hensch T. K. (2002). Permissive proteolytic activity for visual cortical plasticity. *Proc. Natl. Acad. Sci. U.S.A.* 99 7717–7721. 10.1073/pnas.102088899 12032349 PMC124331

[B53] McRae P. A. Porter B. E. (2012). The perineuronal net component of the extracellular matrix in plasticity and epilepsy. *Neurochem. Int.* 61 963–972. 10.1016/j.neuint.2012.08.007 22954428 PMC3930202

[B54] Metropolis N. Rosenbluth A. W. Rosenbluth M. N. Teller A. H. Teller E. (1953). Equation of state calculations by fast computing machines. *J. Chem. Phys.* 21 1087–1092. 10.1063/1.1699114 41342507

[B55] Miller K. D. (1992). Development of orientation columns via competition between ON- and OFF-center inputs. *Neuroreport* 3 73–76. 10.1097/00001756-199201000-00019 1611038

[B56] Miller K. D. (1994). A model for the development of simple cell receptive fields and the ordered arrangement of orientation columns through the activity dependent competition between ON- and OFF-center inputs. *J. Neurosci.* 14 409–441. 10.1523/jneurosci.14-01-00409.1994 8283248 PMC6576834

[B57] Miller K. D. Mackay D. J. (1994). The roles of constraints in Hebbian learning. *Neural Comput.* 6 100–126. 10.1162/neco.1994.6.1.100 32495221

[B58] Miyashita M. Kim D. S. Tanaka S. (1997). Cortical directional selectivity without directional experience. *Neuroreport* 8 1187–1191. 10.1097/00001756-199703240-00026 9175111

[B59] Miyashita M. Tanaka S. (1992). A mathematical model for the self-organization of orientation columns in visual cortex. *Neuroreport* 3 69–72. 10.1097/00001756-199201000-00018 1611037

[B60] Movshon J. A. Thompson I. D. Tolhurst D. J. (1978). Spatial and temporal contrast sensitivity of neurones in areas 17 and 18 of the cat’s visual cortex. *J. Physiol.* 283 101–120. 10.1113/jphysiol.1978.sp012490 722570 PMC1282767

[B61] Müller C. Griesinger C. (1998). Tissue plasminogen activator mediates reverse occlusion plasticity in visual cortex. *Nat. Neurosci.* 1 47–53. 10.1038/248 10195108

[B62] Nagappan G. Zaitsev E. Senatorov V. V. Jr. Yang J. Hempstead B. L. Lu B. (2009). Control of extracellular cleavage of proBDNF by high frequency neuronal activity. *Proc. Natl. Acad. Sci. U.S.A.* 106 1267–1272. 10.1073/pnas.0807322106 19147841 PMC2633536

[B63] Nevian T. Sakmann B. (2004). Single spine Ca2+ signals evoked by coincident EPSPs and backpropagating action potentials in spiny stellate cells of layer 4 in the juvenile rat somatosensory barrel cortex. *J. Neurosci.* 24 1689–1699. 10.1523/jneurosci.3332-03.2004 14973235 PMC6730461

[B64] Numakawa T. Suzuki S. Kumamaru E. Adachi N. Richards M. Kunugi H. (2010). BDNF function and intracellular signaling in neurons. *Histol. Histopathol.* 25 237–258. 20017110 10.14670/HH-25.237

[B65] Obermayer K. Ritter H. Schulten K. (1990). A principle for the formation of the spatial structure of cortical feature maps. *Proc. Nad. Acad. Sci. U.S.A.* 87 8345–8349. 10.1073/pnas.87.21.8345 2236045 PMC54952

[B66] Ohki K. Matsuda Y. Ajima A. Kim D.-S. Tanaka S. (2000). Arrangement of orientation pinwheel centers around area 17/18. *Cereb. Cortex* 10 593–601. 10.1093/cercor/10.6.593 10859137

[B67] Olson C. R. Freeman R. D. (1980). Profile of the sensitive period for monocular deprivation in kittens. *Exp. Brain Res.* 39 17–21. 7379883 10.1007/BF00237065

[B68] Pang T. P. Teng H. K. Zaitsev E. Woo N. T. Sakata K. Zhen S. (2004). Cleavage of proBDNF by tPA/plasmin is essential for long-term hippocampal plasticity. *Science* 306 487–491. 10.1126/science.1100135 15486301

[B69] Rauschecker J. P. Singer W. (1981). The effects of early visual experience on the cat’s visual cortex and their possible explanation by Hebb synapses. *J. Neurophysiol.* 310 215–239. 10.1113/jphysiol.1981.sp013545 7230034 PMC1274736

[B70] Reiter H. O. Stryker M. P. (1988). Neural plasticity without postsynaptic action potentials: Less-active inputs become dominant when kitten visual cortical cells are pharmacologically inhibited. *Proc. Natl. Acad. Sci. U.S.A.* 85 3623–3627. 10.1073/pnas.85.10.3623 3285347 PMC280266

[B71] Saul A. B. Humphrey A. L. (1990). Spatial and temporal response properties of lagged and nonlagged cells in cat lateral geniculate nucleus. *J. Neurophysiol.* 64 206–224. 10.1152/jn.1990.64.1.206 2388066

[B72] Saul A. B. Humphrey A. L. (1992). Evidence of input from lagged cells in the lateral geniculate nucleus to simple cells in cortical area 17 of the cat. *J. Neurophysiol.* 68 1190–1208. 10.1152/jn.1992.68.4.1190 1432077

[B73] Sengpiel F. Stawinski P. Bonhoeffer T. (1999). Influence of experience on orientation maps in cat visual cortex. *Nat. Neurosci.* 2 727–732. 10.1038/11192 10412062

[B74] Shatz C. J. Stryker M. P. (1978). Ocular dominance columns in layer IV of the cat’s visual cortex and the effects of monocular deprivation. *J. Physiol.* 281 267–283. 10.1113/jphysiol.1978.sp012421 702379 PMC1282696

[B75] Singh K. Park K. Hong E. Kramer B. M. Greenberg M. E. Kaplan D. R. (2008). Developmental axon pruning mediated by BDNF-p75NTR-dependent axon degeneration. *Nat. Neurosci.* 11 649–658. 10.1038/nn.2114 18382462

[B76] Stryker M. P. Sherk H. (1975). Modification of cortical selectivity in the cat by restricted visual experience: a reexamination. *Science* 190 904–906. 10.1126/science.1188372 1188372

[B77] Stryker M. P. Sherk H. Leventhal A. G. Hirsch H. V. R. (1978). Physiological consequences for the cat’s visual cortex of effectively restricting early visual experience with oriented contours. *J. Neurophyiol.* 41 894–909. 10.1152/jn.1978.41.4.896 681993

[B78] Sun Y. Lim Y. Li F. Liu S. Lu J.-J. Haberberger R. (2012). ProBDNF collapses neurite outgrowth of primary neurons by activating RhoA. *PLoS One* 7:e35883. 10.1371/journal.pone.0035883 22558255 PMC3338794

[B79] Swindale N. V. (1988). Role of visual experience in promoting segregation of eye dominance patches in the visual cortex of the cat. *J. Comp. Neurol.* 267 472–488. 10.1002/cne.902670403 3346371

[B80] Swindale N. V. Matsubara J. A. Cynader M. S. (1987). Surface organization of orientation and direction selectivity in cat area 18. *J. Neurosci.* 7 1414–1427. 10.1523/jneurosci.07-05-01414.1987 3572486 PMC6568808

[B81] Tanaka S. (1990). Theory of self-organization of cortical maps: mathematical framework. *Neural Netw.* 3 625–640. 10.1016/0893-6080(90)90053-n

[B82] Tanaka S. Miyashita M. (2009). Constraint on the number of synaptic inputs to a visual cortical neuron controls receptive field formation. *Neural. Comput.* 21, 2554–2580. 10.1162/neco.2009.04-08-752 19548800

[B83] Tanaka S. Miyashita M. Ribot J. (2004). Roles of visual experience and intrinsic mechanism in the activity-dependent self-organization of orientation maps: theory and experiment. *Neural Netw.* 17 1363–1375. 10.1016/j.neunet.2004.06.014 15555871

[B84] Tanaka S. Ribot J. Imamura K. Tani T. (2006). Orientation-restricted continuous visual exposure induces marked reorganization of orientation maps in early life. *Neuroimage* 30 462–477. 10.1016/j.neuroimage.2005.09.056 16275019

[B85] Tanaka S. Tani T. Ribot J. O’Hashi K. Imamura K. (2009). A postnatal critical period for orientation plasticity in the cat visual cortex. *PLoS One* 4:e5380 10.1371/journal.pone.005380 PMC2671604 19401781

[B86] Tanaka S. Tani T. Ribot J. Yamazaki T. (2007). Chronically mountable goggles for persistent exposure to single orientation. *J. Neurosci. Methods* 160 206–214. 10.1016/j.jneumeth.2006.09.004 17046067

[B87] Teng H. K. Teng K. K. Lee R. Wright S. Tevar S. Almeida R. D. (2005). ProBDNF induces neuronal apoptosis via activation of a receptor complex of p75NTR and sortilin. *J. Neurosci.* 25 5455–5463. 10.1523/jneurosci.5123-04.2005 15930396 PMC6724992

[B88] Tsai S. J. (2017). Role of tissue-type plasminogen activator and plasminogen activator inhibitor-1 in psychological stress and depression. *Oncotarget* 8 113258–113268. 10.18632/oncotarget.19935 29348904 PMC5762589

[B89] Tsunoda K. Yamane Y. Nishizaki M. Tanifuji M. (2001). Complex objects are represented in macaque inferotemporal cortex by the combination of feature columns. *Nat. Neurosci.* 4 832–838. 10.1038/90547 11477430

[B90] Tusa R. J. Palmer L. A. Rosenquist A. C. (1978). The retinotopic organization of area 17 (striate cortex) in the cat. *J. Comp. Neurol.* 177 213–236. 413845 10.1002/cne.901770204

[B91] Tusa R. J. Rosenquist A. C. Palmer L. A. (1979). Retinotopic organization of areas 18 and 19 in the cat. *J. Comp. Neurol.* 185 657–678. 10.1002/cne.901850405 447876

[B92] Wiesel T. N. Hubel D. H. (1974). Ordered arrangement of orientation columns in monkeys lacking visual experience. *J. Comp. Neurol.* 158 307–318. 10.1002/cne.901580306 4215829

[B93] Yang B. Yang C. Ren Q. Zhang J. C. Chen Q. X. Shirayama Y. (2016). Regional differences in the expression of brain-derived neurotrophic factor (BDNF) pro-peptide, proBDNF and pre-proBDNF in the brain confer stress resilience. *Eur. Arch. Psychiatry Clin. Neurosci.* 266 765–769. 10.1007/s00406-016-0693-6 27094192

[B94] Yokoo T. Knight B. W. Sirovich L. (2001). An optimization approach to signal extraction from noisy multivariate data. *Neuroimage* 14 1309–1326. 10.1006/nimg.2001.0950 11707087

