# Erratum: Development and Reorganization of Orientation Representation in the Cat Visual Cortex: Experience-Dependent Synaptic Rewiring in Early Life

**DOI:** 10.3389/fninf.2020.607901

**Published:** 2020-10-29

**Authors:** Frontiers Production Office

**Affiliations:** Frontiers Media SA, Lausanne, Switzerland

**Keywords:** orientation maps, self-organization, visual experience, development, sensitive period

Due to a production error when converting the original pdf version into other formats the equations included notation errors (zeta–>sigma). These are Equations (7), (8), (13), (14), (16), (16′), and (13′). In addition, there are several notation errors in the sentences around those equations.

The publisher apologizes for this mistake. The original article has been updated.

