# Supplementary material for: Development and Reorganization of Orientation Representation in the Cat Visual Cortex: Experience-Dependent Synaptic Rewiring in Early Life
Source: Front Neuroinform. 2020 Aug 20;14:41. doi: 10.3389/fninf.2020.00041 (PMC7468406; doi:10.3389/fninf.2020.00041)
Supplement: Supplementary file 1 [file Image_1.pdf]

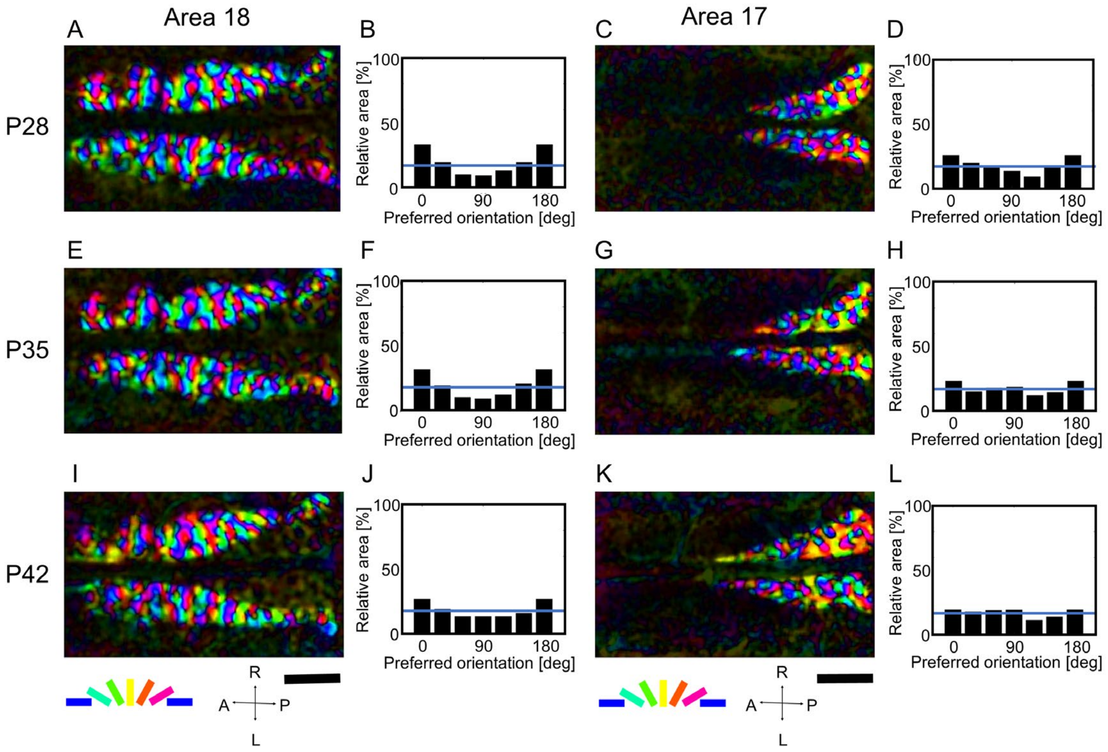

Supplementary Figure 1. An example of the longitudinal maturation of orientation representation in areas 17 and 18 of a kitten reared under normal visual conditions.

(A) and (B), (E) and (F), and (I) and (J): Orientation polar maps and orientation histograms in presumed area 18 at P28, P35, and P42, respectively. (C) and (D), (G) and (H), and (K) and (L): Orientation polar maps and orientation histograms in presumed area 17 at P28, P35, and P42, respectively. In orientation polar maps, preferred orientations are color coded. The orientation magnitude is indicated by the brightness. The scale bar shown below indicates 4 mm. In orientation histograms, the bar height indicates the relative area of iso-orientation domains. The horizontal blue line shows the average relative area in the case of uniform orientation representation. A: anterior, P: posterior, R: right, and L: left in the coordinate system. The orientation polar maps reconstructed using the spatial frequency of 0.15 cpd (A, E, and I) appeared in anatomically presumed area 18 (Tusa et al., 1979). Likewise, orientation polar maps reconstructed using the spatial frequency of 0.5 cpd (C, G, and K) appeared in anatomically presumed area 17 (Tusa et al., 1978). It is noteworthy that areas 17 and 18 were successfully identified using the spatial frequencies of 0.5 and 0.15 cpd from P28 and later, because of clear spatial separation between the two areas. This areal identification provides a basis of research on the sensitive period profile in area 17. The histograms (B, F, and J) showed distributions

extremely biased towards horizontal orientation, indicating that the relative area of responsive domains in area 18 was largest at horizontal orientation (0º or equivalently 180º) and smallest at vertical orientation (90º). Innate orientation bias was also seen in area 17, although it was weaker than in area 18 (D, H, and L). The strong innate orientation bias in area 18 makes the analysis for the sensitive period profile difficult. Due to this fact, we focused on orientation representation in area 17 in this study. In addition, to avoid complexity originating from orientation bias towards horizontal orientation, we analyzed data from kittens exposed to only vertical orientation to obtain the sensitive period profile.
