# Supplementary material for: Development and Reorganization of Orientation Representation in the Cat Visual Cortex: Experience-Dependent Synaptic Rewiring in Early Life
Source: Front Neuroinform. 2020 Aug 20;14:41. doi: 10.3389/fninf.2020.00041 (PMC7468406; doi:10.3389/fninf.2020.00041)
Supplement: Supplementary file 2 [file Image_2.pdf]

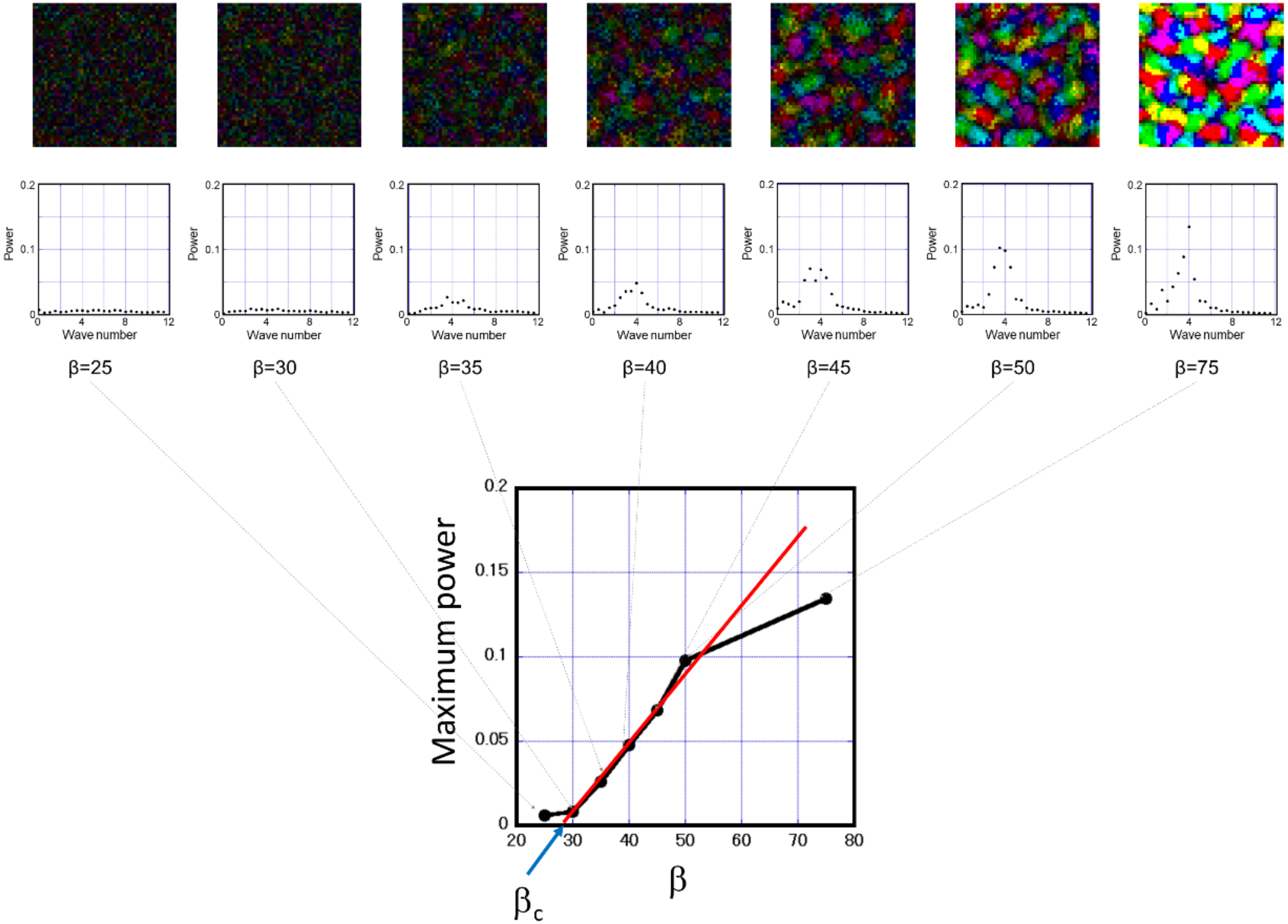

Supplementary Figure 2. Phase transition of orientation representation.

Orientation representation undergoes a sort of phase transition at $\beta = \beta_c$ from the random representation phase ($\beta < \beta_c$) to the orderly orientation map phase ($\beta > \beta_c$), when the value of the inverse temperature β increases from 0. The top row shows the orientation representations at different values of β. The middle row indicates the radial power spectra of respective orientation representations. The bottom figure illustrates the β-dependence of the maximum power. The maximum power changes almost linearly within the interval of $30 \leq \beta \leq 45$, as indicated by the red line. The transition point β_c is reasonably determined by the intersection of the red line with the horizontal axis, and we obtain $\beta_c \approx 28$ in the parameter setting of simulations that we performed in this study.
