# Supplementary material for: Development and Reorganization of Orientation Representation in the Cat Visual Cortex: Experience-Dependent Synaptic Rewiring in Early Life
Source: Front Neuroinform. 2020 Aug 20;14:41. doi: 10.3389/fninf.2020.00041 (PMC7468406; doi:10.3389/fninf.2020.00041)
Supplement: Supplementary file 3 [file Image_3.pdf]

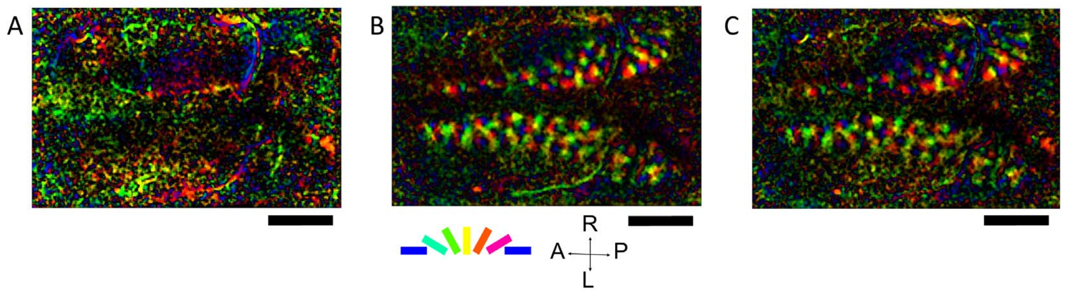

Supplementary Figure 3. Zero-power lens effects

A kitten was reared with a transparent zero-power lens in the right eye and an opaque zero-power lens in the left eye from P22 to P107. Optical imaging in the visual cortex was conducted at P108 for left-eye stimulation, right-eye stimulation, and binocular stimulation of 6 sinusoidal oriented gratings moving back and forth in the directions orthogonal to the stimulus orientations. The temporal and spatial frequencies were 2Hz and 0.5 cpd, respectively. (A) The absence of orientation representation for the left-eye stimulation. (B) Orientation map reconstructed by the right-eye stimulation, (C) Orientation map reconstructed for binocular stimulation.

Figure 3A indicates that no orientation map was formed in the kitten exposed to light but deprived of the experience of visual environment. This observation implies that the formation or maintenance of orientation maps requires pattern vision rather than simple exposure to light. Figure 3B suggests that the transparent zero-power lens does not interfere with the formation of a regular orientation map. It is also demonstrated that the marked over-representation of the exposed orientation cannot take place accidentally or spontaneously without nonzero-power lenses.
