# Supplementary material for: Development and Reorganization of Orientation Representation in the Cat Visual Cortex: Experience-Dependent Synaptic Rewiring in Early Life
Source: Front Neuroinform. 2020 Aug 20;14:41. doi: 10.3389/fninf.2020.00041 (PMC7468406; doi:10.3389/fninf.2020.00041)
Supplement: Supplementary file 4 [file Image_4.pdf]

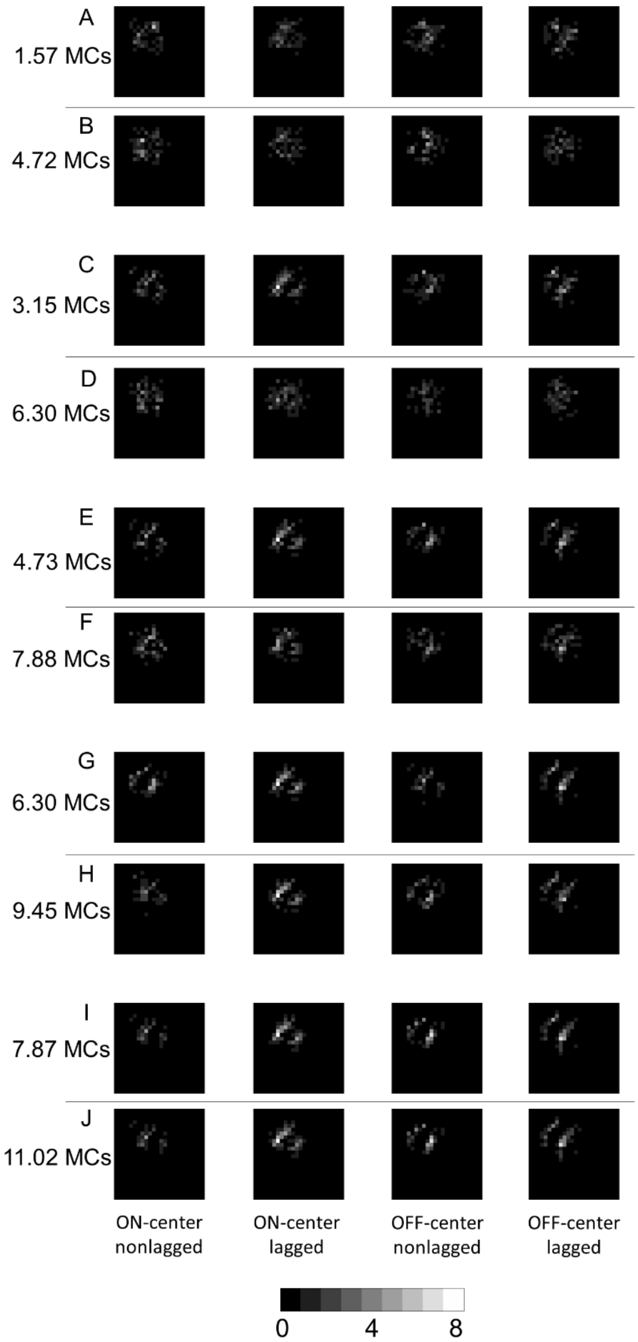

Supplementary Figure 4. Spatial patterns of four types of afferent synaptic inputs.

Preferred orientations are determined by the spatial arrangement of four types of inputs. Spatiotemporal receptive field are calculated by the convolution with LGN spatiotemporal receptive fields. Cortical spatiotemporal receptive fields shown in Fig. 11 are obtained from these synaptic inputs. The gradation indicates the number of inputs. The white indicates 8 synaptic inputs or more, whereas the black indicates 0 input.
