# Supplementary material for: Development and Reorganization of Orientation Representation in the Cat Visual Cortex: Experience-Dependent Synaptic Rewiring in Early Life
Source: Front Neuroinform. 2020 Aug 20;14:41. doi: 10.3389/fninf.2020.00041 (PMC7468406; doi:10.3389/fninf.2020.00041)
Supplement: Supplementary file 5 [file Table_1.docx]

Supplementary Table 1. Orientation representations and orientation selectivity index in 2-week goggle-reared cats

| Cat ID | Age of GR onset [day] | Age of optical imaging [day] | Relative areas of orientation representations [%] | | | | | | OSI | |
| --- | --- | --- | --- | --- | --- | --- | --- | --- | --- | --- |
|  |  |  | 0 deg | 30 deg | 60 deg | 90 deg | 120 deg | 150 deg | Peak | Average |
| GFa1 | 10 | 24 | 29.16 | 13.34 | 12.42 | 17.939 | 9.66 | 17.48 | 0.231 | 0.231 |
| FUc1 | 17 | 31 | 3.47 | 5.39 | 11.06 | 57.13 | 17.00 | 5.94 | 0.413 | 0.307 |
| HYb1 | 21 | 35 | 1.20 | 1.85 | 8.50 | 66.64 | 17.56 | 4.25 | 0.427 | 0.302 |
| FOb2 | 22 | 36 | 2.93 | 4.77 | 10.91 | 58.66 | 17.14 | 5.59 | 0.427 | 0.314 |
| GFb2 | 23 | 37 | 1.65 | 2.57 | 9.91 | 77.16 | 6.88 | 1.83 | 0.511 | 0.386 |
| HYc1 | 24 | 38 | 1.28 | 1.28 | 6.87 | 87.18 | 2.75 | 0.64 | 0.518 | 0.416 |
| FUd1 | 27 | 41 | 0.46 | 0.92 | 10.04 | 78.64 | 8.29 | 1.66 | 0.532 | 0.402 |
| HBa3 | 31 | 45 | 6.09 | 4.26 | 8.89 | 68.20 | 8.09 | 4.47 | - | - |
| GKe1 | 32 | 46 | 3.21 | 2.84 | 9.71 | 74.54 | 6.32 | 3.39 | 0.616 | 0.478 |
| HZa1 | 34 | 48 | 5.68 | 5.13 | 10.99 | 47.80 | 23.08 | 7.326 | - | - |
| FTa1 | 37 | 51 | 8.66 | 7.37 | 10.13 | 50.83 | 12.80 | 10.22 | 0.497 | 0.357 |
| HZb1 | 39 | 53 | 1.83 | 3.66 | 12.36 | 66.03 | 12.91 | 3.21 | 0.434 | 0.309 |
| FQd1 | 40 | 54 | 5.60 | 6.88 | 17.16 | 37.06 | 25.69 | 7.61 | 0.252 | 0.252 |
| IBb1 | 45 | 59 | 5.33 | 10.19 | 26.81 | 37.19 | 14.88 | 5.60 | 0.301 | 0.271 |
| FOc1 | 54 | 68 | 23.40 | 22.39 | 13.21 | 11.93 | 12.02 | 17.06 | 0.42 | 0.343 |
| IAb2 | 73 | 87 | 21.33 | 23.72 | 16.95 | 8.42 | 13.74 | 15.84 | 0.42 | 0.319 |

About Cat ID: A pair of capital letters, lower-case letter, and numeral indicate a mother cat, kitten from the same litter, and the number of recording, respectively. In cats HBa3 and HZa1, the data on OSI were missing. Data in the hatched cells were used to estimate means and standard errors (SE) in Figure 5.
