# Supplementary material for: Development and Reorganization of Orientation Representation in the Cat Visual Cortex: Experience-Dependent Synaptic Rewiring in Early Life
Source: Front Neuroinform. 2020 Aug 20;14:41. doi: 10.3389/fninf.2020.00041 (PMC7468406; doi:10.3389/fninf.2020.00041)
Supplement: Supplementary file 6 [file Table_2.docx]

Supplementary Table 2. Relative areas of orientation representation for short-term single-orientation exposure and normal viewing

| Table 2-1. Normal rearing | | | | | | | |  |
| --- | --- | --- | --- | --- | --- | --- | --- | --- |
| Cat ID |  | Age of optical imaging [day] | Relative areas of orientation representations [%] | | | | | |
|  |  |  | 0 deg | 30 deg | 60 deg | 90 deg | 120 deg | 150 deg |
| GVa1 | - | 29 | 20.8408 | 22.3399 | 15.5402 | 13.4509 | 12.3133 | 15.5148 |
| GYe1 | - | 29 | 24.7702 | 14.0914 | 8.55568 | 13.5423 | 16.971 | 22.0695 |
| GZb1 | - | 29 | 36.7758 | 23.2231 | 7.22626 | 4.73516 | 10.257 | 17.7827 |
| HBb1 | - | 29 | 33.6604 | 15.0652 | 9.13471 | 11.5789 | 11.0812 | 19.4796 |
|  |  |  |  |  |  |  |  |  |
| Table 2-2. 1-w Goggle rearing | | | | | | | | |
| Cat ID | Age of GR onset [day] | Age of optical imaging [day] | Relative areas of orientation representations [%] | | | | | |
|  |  |  | 0 deg | 30 deg | 60 deg | 90 deg | 120 deg | 150 deg |
| GXa3 | 32 | 39 | 2.78278 | 2.22409 | 5.1122 | 82.7935 | 4.4436 | 2.64387 |
| GYd3 | 32 | 41 | 5.50379 | 5.40554 | 7.35703 | 71.4748 | 8.63938 | 1.61946 |
| GYe3 | 34 | 42 | 12.4255 | 7.46005 | 15.4346 | 46.152 | 8.60706 | 9.92068 |
| GSb2 | 31 | 38 | 7.79888 | 9.90079 | 14.1923 | 37.396 | 21.1619 | 9.55006 |
| HBa3 | 31 | 38 | 8.90929 | 7.4465 | 13.6237 | 50.2042 | 12.5119 | 7.3044 |
| HEa3 | 30 | 40 | 18.7326 | 11.7302 | 14.2854 | 28.8083 | 12.3992 | 14.0442 |
|  |  |  |  |  |  |  |  |  |
| Table 2-3. Normal viewing | | | | | | | | |
| Cat ID | Age of goggle removal [day] | Age of optical imaging [day] | Relative areas of orientation representations [%] | | | | | |
|  |  |  | 0 deg | 30 deg | 60 deg | 90 deg | 120 deg | 150 deg |
| GYe4 | 42 | 49 | 16.5677 | 17.8316 | 17.0249 | 17.2036 | 16.1144 | 15.2578 |
| GYd4 | 41 | 48 | 14.614 | 17.7855 | 17.4046 | 17.8844 | 15.9275 | 16.384 |
| GZb4 | 42 | 49 | 14.9797 | 15.1287 | 18.0308 | 19.5633 | 16.1178 | 16.1797 |
| HEa4 | 40 | 47 | 15.7236 | 16.725 | 18.8374 | 18.7211 | 15.696 | 14.2315 |
| HFa1 | 37 | 50 | 19.7236 | 14.8405 | 12.9859 | 14.4366 | 17.2461 | 20.7673 |
