# Supplementary material for: Development and Reorganization of Orientation Representation in the Cat Visual Cortex: Experience-Dependent Synaptic Rewiring in Early Life
Source: Front Neuroinform. 2020 Aug 20;14:41. doi: 10.3389/fninf.2020.00041 (PMC7468406; doi:10.3389/fninf.2020.00041)
Supplement: Supplementary file 7 [file Table_3.docx]

Supplementary Table 3. Relative areas of orientation representation for prolonged goggle rearing and successive normal viewing

| Cat ID | Age of GR onset [day] | Age of goggle removal [day] |  | Age of optical imaging [day] | Relative areas of orientation representations [%] | | | | | |
| --- | --- | --- | --- | --- | --- | --- | --- | --- | --- | --- |
|  |  |  |  |  | 0 deg | 30 deg | 60 deg | 90 deg | 120 deg | 150 deg |
| GSc1 | 24 | - |  | 73 | 13.28 | 11.81 | 22.20 | 30.51 | 12.03 | 10.17 |
| GSc4 | - | 73 |  | 130 | 15.89 | 10.00 | 11.62 | 28.97 | 19.76 | 13.76 |
| GSd1 | 25 | - |  | 74 | 15.96 | 4.74 | 9.87 | 55.84 | 9.42 | 4.17 |
| GSd3 | - | 74 |  | 95 | 7.70 | 3.65 | 6.57 | 58.37 | 17.87 | 5.84 |
| HTa1 | 24 | - |  | 109 | 23.51 | 2.71 | 6.20 | 44.14 | 12.85 | 10.60 |
| HTa3 | - | 109 |  | 143 | 22.40 | 5.98 | 8.18 | 36.12 | 13.21 | 14.11 |
