# Supplementary material for: Development and Reorganization of Orientation Representation in the Cat Visual Cortex: Experience-Dependent Synaptic Rewiring in Early Life
Source: Front Neuroinform. 2020 Aug 20;14:41. doi: 10.3389/fninf.2020.00041 (PMC7468406; doi:10.3389/fninf.2020.00041)
Supplement: Supplementary file 8 [file Table_4.docx]

Supplementary Table 4. Numerical data of the relative number of neurons for six preferred orientations at five trials at different Monte Carlo steps of simulations performed under balanced exposure to 12 stimulus orientations.

| Table 4-1. N=0 | | | | | | | |
| --- | --- | --- | --- | --- | --- | --- | --- |
| Preferred orientation | trial1 | trial2 | trial3 | trial4 | trial5 | mean | SE |
| 0 | 0.1849 | 0.16667 | 0.16016 | 0.15712 | 0.16753 | 0.16727 | 0.004821 |
| 30 | 0.18012 | 0.16059 | 0.16363 | 0.17361 | 0.17795 | 0.17118 | 0.003879 |
| 60 | 0.15017 | 0.16667 | 0.17274 | 0.1671 | 0.17578 | 0.16649 | 0.004428 |
| 90 | 0.16536 | 0.17795 | 0.17491 | 0.16884 | 0.15495 | 0.1684 | 0.004025 |
| 120 | 0.16319 | 0.16363 | 0.1697 | 0.16146 | 0.15929 | 0.16345 | 0.001739 |
| 150 | 0.15625 | 0.1645 | 0.15885 | 0.17188 | 0.1645 | 0.16319 | 0.0027 |
|  |  |  |  |  |  |  |  |
|  |  |  |  |  |  |  |  |
| Table 4-2. N=0.2 | | | | | | | |
| Preferred orientation | trial1 | trial2 | trial3 | trial4 | trial5 | mean | SE |
| 0 | 0.16797 | 0.17925 | 0.18142 | 0.16059 | 0.16927 | 0.1717 | 0.00384 |
| 30 | 0.17405 | 0.16797 | 0.16363 | 0.17057 | 0.16189 | 0.16762 | 0.002223 |
| 60 | 0.1671 | 0.16102 | 0.18186 | 0.16667 | 0.18229 | 0.17179 | 0.004335 |
| 90 | 0.15972 | 0.17144 | 0.16319 | 0.17405 | 0.1645 | 0.16658 | 0.002666 |
| 120 | 0.15799 | 0.17318 | 0.16623 | 0.16884 | 0.15061 | 0.16337 | 0.004038 |
| 150 | 0.17318 | 0.14714 | 0.14366 | 0.15929 | 0.17144 | 0.15894 | 0.006049 |
|  |  |  |  |  |  |  |  |
|  |  |  |  |  |  |  |  |
| Table 4-3. N=0.39 | | | | | | | |
| Preferred orientation | trial1 | trial2 | trial3 | trial4 | trial5 | mean | SE |
| 0 | 0.1645 | 0.17361 | 0.17795 | 0.16233 | 0.1645 | 0.16858 | 0.003046 |
| 30 | 0.17925 | 0.17231 | 0.17448 | 0.16753 | 0.16667 | 0.17205 | 0.002315 |
| 60 | 0.16363 | 0.15842 | 0.16536 | 0.17057 | 0.17882 | 0.16736 | 0.00346 |
| 90 | 0.15408 | 0.17231 | 0.16059 | 0.17795 | 0.1645 | 0.16589 | 0.00422 |
| 120 | 0.16363 | 0.16536 | 0.16189 | 0.15799 | 0.15234 | 0.16024 | 0.002323 |
| 150 | 0.17491 | 0.15799 | 0.15972 | 0.16363 | 0.17318 | 0.16589 | 0.003465 |

| Table 4-4. N=0.79 | | | | | | | |
| --- | --- | --- | --- | --- | --- | --- | --- |
| Preferred orientation | trial1 | trial2 | trial3 | trial4 | trial5 | mean | SE |
| 0 | 0.15408 | 0.17665 | 0.17795 | 0.19705 | 0.17144 | 0.17543 | 0.006881 |
| 30 | 0.18186 | 0.16276 | 0.17101 | 0.16753 | 0.1697 | 0.17057 | 0.003151 |
| 60 | 0.17491 | 0.16363 | 0.15365 | 0.17708 | 0.17578 | 0.16901 | 0.004533 |
| 90 | 0.16189 | 0.17752 | 0.17057 | 0.16623 | 0.17361 | 0.16997 | 0.002737 |
| 120 | 0.15929 | 0.15191 | 0.16493 | 0.16189 | 0.14149 | 0.1559 | 0.004198 |
| 150 | 0.16797 | 0.17188 | 0.16189 | 0.17361 | 0.16797 | 0.16866 | 0.00202 |
|  |  |  |  |  |  |  |  |
|  |  |  |  |  |  |  |  |
| Table 4-5. N=1.58 | | | | | | | |
| Preferred orientation | trial1 | trial2 | trial3 | trial4 | trial5 | mean | SE |
| 0 | 0.15278 | 0.18186 | 0.19271 | 0.16233 | 0.16406 | 0.17075 | 0.007226 |
| 30 | 0.1671 | 0.15148 | 0.16406 | 0.15885 | 0.15191 | 0.15868 | 0.003144 |
| 60 | 0.17665 | 0.17274 | 0.15712 | 0.17448 | 0.18316 | 0.17283 | 0.004306 |
| 90 | 0.15278 | 0.17231 | 0.18316 | 0.18967 | 0.16276 | 0.17214 | 0.006677 |
| 120 | 0.17795 | 0.14193 | 0.14366 | 0.14497 | 0.14974 | 0.15165 | 0.006702 |
| 150 | 0.17274 | 0.17969 | 0.15929 | 0.1697 | 0.18837 | 0.17396 | 0.004875 |
|  |  |  |  |  |  |  |  |
|  |  |  |  |  |  |  |  |
| Table 4-6. N=2.36 | | | | | | | |
| Preferred orientation | trial1 | trial2 | trial3 | trial4 | trial5 | mean | SE |
| 0 | 0.16059 | 0.17622 | 0.19488 | 0.17144 | 0.1671 | 0.17405 | 0.005809 |
| 30 | 0.16797 | 0.15017 | 0.16536 | 0.15929 | 0.14887 | 0.15833 | 0.003869 |
| 60 | 0.17188 | 0.18012 | 0.15278 | 0.17622 | 0.1862 | 0.17344 | 0.005677 |
| 90 | 0.15148 | 0.17491 | 0.18967 | 0.17925 | 0.1684 | 0.17274 | 0.006343 |
| 120 | 0.18446 | 0.13411 | 0.13932 | 0.1454 | 0.1428 | 0.14922 | 0.009011 |
| 150 | 0.16363 | 0.18446 | 0.15799 | 0.1684 | 0.18663 | 0.17222 | 0.005695 |
|  |  |  |  |  |  |  |  |

| Table 4-7. N=3.15 | | | | | | | |
| --- | --- | --- | --- | --- | --- | --- | --- |
| Preferred orientation | trial1 | trial2 | trial3 | trial4 | trial5 | mean | SE |
| 0 | 0.1697 | 0.17405 | 0.20312 | 0.17318 | 0.16059 | 0.17613 | 0.007157 |
| 30 | 0.16363 | 0.15451 | 0.16363 | 0.16797 | 0.14714 | 0.15937 | 0.003765 |
| 60 | 0.17188 | 0.17882 | 0.14714 | 0.1697 | 0.1849 | 0.17049 | 0.006422 |
| 90 | 0.15495 | 0.17318 | 0.19531 | 0.18359 | 0.16623 | 0.17465 | 0.006958 |
| 120 | 0.18142 | 0.14149 | 0.13585 | 0.14627 | 0.13932 | 0.14887 | 0.008311 |
| 150 | 0.15842 | 0.17795 | 0.15495 | 0.15929 | 0.20182 | 0.17049 | 0.008804 |
|  |  |  |  |  |  |  |  |
| Table 4-8. N=3.94 | | | | | | | |
| Preferred orientation | trial1 | trial2 | trial3 | trial4 | trial5 | mean | SE |
| 0 | 0.16493 | 0.17405 | 0.20182 | 0.17318 | 0.15104 | 0.173 | 0.008304 |
| 30 | 0.16276 | 0.15755 | 0.1684 | 0.16536 | 0.15017 | 0.16085 | 0.003209 |
| 60 | 0.1684 | 0.17969 | 0.14757 | 0.16927 | 0.18663 | 0.17031 | 0.006619 |
| 90 | 0.15451 | 0.17144 | 0.19922 | 0.18142 | 0.1645 | 0.17422 | 0.00764 |
| 120 | 0.18316 | 0.14149 | 0.13585 | 0.14627 | 0.13672 | 0.1487 | 0.008815 |
| 150 | 0.16623 | 0.17578 | 0.15495 | 0.1645 | 0.21094 | 0.17448 | 0.009695 |
|  |  |  |  |  |  |  |  |
| Table 4-9. N=4.73 | | | | | | | |
| Preferred orientation | trial1 | trial2 | trial3 | trial4 | trial5 | ave. | SE |
| 0 | 0.16406 | 0.17274 | 0.19661 | 0.1697 | 0.15061 | 0.17075 | 0.007498 |
| 30 | 0.16102 | 0.15929 | 0.1658 | 0.16927 | 0.14844 | 0.16076 | 0.003549 |
| 60 | 0.17188 | 0.17535 | 0.15668 | 0.16927 | 0.19184 | 0.173 | 0.005665 |
| 90 | 0.15148 | 0.17578 | 0.19184 | 0.18316 | 0.16059 | 0.17257 | 0.007356 |
| 120 | 0.1849 | 0.1441 | 0.13194 | 0.14887 | 0.13715 | 0.14939 | 0.009336 |
| 150 | 0.16667 | 0.17274 | 0.15712 | 0.15972 | 0.21137 | 0.17352 | 0.009848 |

| Table 4-10. N=5.51 | | | | | | | |
| --- | --- | --- | --- | --- | --- | --- | --- |
| Preferred orientation | trial1 | trial2 | trial3 | trial4 | trial5 | mean | SE |
| 0 | 0.1658 | 0.17578 | 0.19531 | 0.16102 | 0.15234 | 0.17005 | 0.007362 |
| 30 | 0.15842 | 0.15712 | 0.17014 | 0.17925 | 0.14887 | 0.16276 | 0.005339 |
| 60 | 0.17014 | 0.17318 | 0.15278 | 0.16884 | 0.18967 | 0.17092 | 0.005877 |
| 90 | 0.15191 | 0.17839 | 0.19401 | 0.18056 | 0.16059 | 0.17309 | 0.007504 |
| 120 | 0.1875 | 0.14193 | 0.13021 | 0.14844 | 0.14193 | 0.15 | 0.009825 |
| 150 | 0.16623 | 0.17361 | 0.15755 | 0.16189 | 0.2066 | 0.17318 | 0.008766 |
|  |  |  |  |  |  |  |  |
| Table 4-11. N=6.30 | | | | | | | |
| Preferred orientation | trial1 | trial2 | trial3 | trial4 | trial5 | mean | SE |
| 0 | 0.1671 | 0.17578 | 0.19618 | 0.16189 | 0.15582 | 0.17135 | 0.007018 |
| 30 | 0.15582 | 0.15408 | 0.17014 | 0.17491 | 0.14583 | 0.16016 | 0.005377 |
| 60 | 0.17405 | 0.17882 | 0.15104 | 0.17318 | 0.18924 | 0.17326 | 0.006247 |
| 90 | 0.14931 | 0.17622 | 0.19401 | 0.17839 | 0.1645 | 0.17248 | 0.007461 |
| 120 | 0.19097 | 0.14149 | 0.13151 | 0.14974 | 0.13845 | 0.15043 | 0.010548 |
| 150 | 0.16276 | 0.17361 | 0.15712 | 0.16189 | 0.20616 | 0.17231 | 0.008883 |
|  |  |  |  |  |  |  |  |
| Table 4-12. N=7.09 | | | | | | | |
| Preferred orientation | trial1 | trial2 | trial3 | trial4 | trial5 | mean | SE |
| 0 | 0.16884 | 0.17969 | 0.19358 | 0.15712 | 0.15625 | 0.17109 | 0.007071 |
| 30 | 0.15929 | 0.15408 | 0.1671 | 0.18012 | 0.14583 | 0.16128 | 0.005845 |
| 60 | 0.17188 | 0.17752 | 0.15408 | 0.17101 | 0.18967 | 0.17283 | 0.005751 |
| 90 | 0.14974 | 0.17361 | 0.19401 | 0.17925 | 0.16536 | 0.1724 | 0.007343 |
| 120 | 0.19358 | 0.14366 | 0.13368 | 0.14757 | 0.14106 | 0.15191 | 0.010661 |
| 150 | 0.15668 | 0.17144 | 0.15755 | 0.16493 | 0.20182 | 0.17049 | 0.008281 |

| Table 4-13. N=7.88 | | | | | | | |
| --- | --- | --- | --- | --- | --- | --- | --- |
| Preferred orientation | trial1 | trial2 | trial3 | trial4 | trial5 | mean | SE |
| 0 | 0.16753 | 0.17708 | 0.19358 | 0.16102 | 0.15625 | 0.17109 | 0.006617 |
| 30 | 0.15885 | 0.15712 | 0.1671 | 0.17665 | 0.14627 | 0.1612 | 0.005092 |
| 60 | 0.17318 | 0.17665 | 0.15582 | 0.17188 | 0.18793 | 0.17309 | 0.005162 |
| 90 | 0.14931 | 0.17578 | 0.19488 | 0.17795 | 0.16536 | 0.17266 | 0.007517 |
| 120 | 0.19097 | 0.14106 | 0.13368 | 0.15234 | 0.14106 | 0.15182 | 0.010232 |
| 150 | 0.15929 | 0.17231 | 0.15495 | 0.16016 | 0.18142 | 0.16563 | 0.004891 |
|  |  |  |  |  |  |  |  |
| Table 4-14. N=11.81 | | | | | | | |
| Preferred orientation | trial1 | trial2 | trial3 | trial4 | trial5 | mean | SE |
| 0 | 0.16884 | 0.17925 | 0.19054 | 0.16319 | 0.15365 | 0.17109 | 0.00639 |
| 30 | 0.15538 | 0.15712 | 0.16797 | 0.17491 | 0.14844 | 0.16076 | 0.004724 |
| 60 | 0.17405 | 0.17491 | 0.15625 | 0.17231 | 0.1875 | 0.173 | 0.00498 |
| 90 | 0.14931 | 0.17535 | 0.19358 | 0.17578 | 0.16319 | 0.17144 | 0.007356 |
| 120 | 0.19141 | 0.14453 | 0.13325 | 0.15234 | 0.14019 | 0.15234 | 0.010245 |
| 150 | 0.16102 | 0.16884 | 0.15842 | 0.16146 | 0.20703 | 0.17135 | 0.009086 |
|  |  |  |  |  |  |  |  |
| Table 4-15. N=15.75 | | | | | | | |
| Preferred orientation | trial1 | trial2 | trial3 | trial4 | trial5 | mean | SE |
| 0 | 0.16884 | 0.17882 | 0.19054 | 0.16363 | 0.15321 | 0.17101 | 0.006397 |
| 30 | 0.15538 | 0.15712 | 0.16797 | 0.17491 | 0.14844 | 0.16076 | 0.004724 |
| 60 | 0.17361 | 0.17491 | 0.15625 | 0.17231 | 0.18793 | 0.173 | 0.00504 |
| 90 | 0.15017 | 0.17535 | 0.19358 | 0.17535 | 0.16319 | 0.17153 | 0.007214 |
| 120 | 0.19097 | 0.14453 | 0.13325 | 0.15278 | 0.14019 | 0.15234 | 0.010163 |
| 150 | 0.16102 | 0.16927 | 0.15842 | 0.16102 | 0.20703 | 0.17135 | 0.009105 |
