# Supplementary material for: Development and Reorganization of Orientation Representation in the Cat Visual Cortex: Experience-Dependent Synaptic Rewiring in Early Life
Source: Front Neuroinform. 2020 Aug 20;14:41. doi: 10.3389/fninf.2020.00041 (PMC7468406; doi:10.3389/fninf.2020.00041)
Supplement: Supplementary file 9 [file Table_5.docx]

Supplementary Table 5. Numerical data of the relative number of neurons for six preferred orientations at five trials of simulations under vertical orientation exposure following simulations under balanced exposure to 12 stimulus orientations. N1: Monte Carlo steps of simulation under 12-orientation exposure, N2: Monte Carlo steps of simulation under single-orientation exposure, and N: Total Monte Carlo steps.

Table 5-1. N=0.79 (N1=0, N2=0.79)

| Preferred orientation. | trial1 | trial2 | trial3 | trial4 | trial5 | Mean. | SE |
| --- | --- | --- | --- | --- | --- | --- | --- |
| 0 [deg] | 0.048177 | 0.049045 | 0.045139 | 0.052951 | 0.055556 | 0.050174 | 0.001834 |
| 30 | 0.075955 | 0.078993 | 0.06901 | 0.070747 | 0.074219 | 0.073785 | 0.00179 |
| 60 | 0.20182 | 0.20052 | 0.2092 | 0.20009 | 0.19184 | 0.20069 | 0.002761 |
| 90 | 0.40712 | 0.40799 | 0.39974 | 0.4184 | 0.42101 | 0.41085 | 0.00391 |
| 120 | 0.19271 | 0.19141 | 0.20182 | 0.20226 | 0.18576 | 0.19479 | 0.003182 |
| 150 | 0.074219 | 0.072049 | 0.075087 | 0.055556 | 0.071615 | 0.069705 | 0.003596 |
|  |  |  |  |  |  |  |  |
|  |  |  |  |  |  |  |  |
| Table 5-2. N=1.57 (N1=0, N2=1.57) | | | | | | | |
| Preferred orientation. | trial1 | trial2 | trial3 | trial4 | trial5 | Mean. | SE |
| 0 [deg] | 0.022135 | 0.014757 | 0.011285 | 0.019097 | 0.023003 | 0.018056 | 0.002224 |
| 30 | 0.022135 | 0.03125 | 0.037326 | 0.026476 | 0.03559 | 0.030556 | 0.002822 |
| 60 | 0.16884 | 0.16406 | 0.1671 | 0.16884 | 0.17101 | 0.16797 | 0.001157 |
| 90 | 0.57292 | 0.59332 | 0.6033 | 0.59158 | 0.59071 | 0.59036 | 0.004909 |
| 120 | 0.17448 | 0.1671 | 0.14844 | 0.16189 | 0.15148 | 0.16068 | 0.004836 |
| 150 | 0.036024 | 0.029514 | 0.032552 | 0.032118 | 0.028212 | 0.031684 | 0.001352 |
|  |  |  |  |  |  |  |  |
|  |  |  |  |  |  |  |  |
| Table 5-3. N=2.36 (N1=0, N2=2.36) | | | | | | | |
| Preferred orientation. | trial1 | trial2 | trial3 | trial4 | trial5 | Mean. | SE |
| 0 [deg] | 0.011285 | 0.010417 | 0.015191 | 0.007379 | 0.007379 | 0.01033 | 0.001449 |
| 30 | 0.018229 | 0.017795 | 0.017361 | 0.017795 | 0.016059 | 0.017448 | 0.000373 |
| 60 | 0.13368 | 0.13715 | 0.13542 | 0.13064 | 0.12283 | 0.13194 | 0.00252 |
| 90 | 0.68316 | 0.67839 | 0.67708 | 0.68924 | 0.70747 | 0.68707 | 0.005528 |
| 120 | 0.14106 | 0.13759 | 0.14323 | 0.13845 | 0.12934 | 0.13793 | 0.002367 |
| 150 | 0.012587 | 0.018663 | 0.011719 | 0.016493 | 0.016927 | 0.015278 | 0.001334 |
| Table 5-4. N=3.15 (N1=0, N2=3.15) | | | | | | | |
| Preferred orientation. | trial1 | trial2 | trial3 | trial4 | trial5 | Mean. | SE |
| 0 [deg] | 0.00651 | 0.005642 | 0.008247 | 0.005208 | 0.006076 | 0.006337 | 0.000524 |
| 30 | 0.007813 | 0.008247 | 0.012587 | 0.013021 | 0.007379 | 0.009809 | 0.001232 |
| 60 | 0.10894 | 0.10807 | 0.10286 | 0.1046 | 0.11285 | 0.10747 | 0.001745 |
| 90 | 0.76172 | 0.75998 | 0.74609 | 0.76042 | 0.76302 | 0.75825 | 0.003084 |
| 120 | 0.10417 | 0.10547 | 0.11979 | 0.10851 | 0.10026 | 0.10764 | 0.003314 |
| 150 | 0.010851 | 0.009983 | 0.010417 | 0.008247 | 0.010417 | 0.009983 | 0.000455 |
|  |  |  |  |  |  |  |  |
|  |  |  |  |  |  |  |  |
| Table 5-5. N=3.35 (N1=0.2, N2=3.15) | | | | | | | |
| Preferred orientation. | trial1 | trial2 | trial3 | trial4 | trial5 | Mean. | SE |
| 0 [deg] | 0.00434 | 0.004774 | 0.003906 | 0.004774 | 0.008247 | 0.005208 | 0.000776 |
| 30 | 0.009115 | 0.011719 | 0.010417 | 0.009549 | 0.010417 | 0.010243 | 0.000447 |
| 60 | 0.11806 | 0.10156 | 0.11198 | 0.11024 | 0.10677 | 0.10972 | 0.00274 |
| 90 | 0.7526 | 0.75391 | 0.75998 | 0.75347 | 0.75434 | 0.75486 | 0.001312 |
| 120 | 0.10634 | 0.11241 | 0.10113 | 0.11285 | 0.10981 | 0.10851 | 0.002179 |
| 150 | 0.009549 | 0.015625 | 0.009983 | 0.009115 | 0.010417 | 0.010937 | 0.001192 |
|  |  |  |  |  |  |  |  |
|  |  |  |  |  |  |  |  |
| Table 5-6. N=3.54 (N1=0.39, N2 = 3.15) | | | | | | | |
| Preferred orientation. | trial1 | trial2 | trial3 | trial4 | trial5 | Mean. | SE |
| 0 [deg] | 0.003906 | 0.003038 | 0.003472 | 0.006076 | 0.005642 | 0.004427 | 0.000605 |
| 30 | 0.009115 | 0.015191 | 0.012153 | 0.009549 | 0.009549 | 0.011111 | 0.001153 |
| 60 | 0.10417 | 0.1046 | 0.11632 | 0.11415 | 0.10938 | 0.10972 | 0.002453 |
| 90 | 0.77257 | 0.76953 | 0.75217 | 0.75651 | 0.75868 | 0.76189 | 0.003913 |
| 120 | 0.10417 | 0.097222 | 0.10503 | 0.10547 | 0.10503 | 0.10339 | 0.001555 |
| 150 | 0.006076 | 0.010417 | 0.010851 | 0.008247 | 0.011719 | 0.009462 | 0.001022 |
|  |  |  |  |  |  |  |  |

| Table 5-7. N=3.94 (N1 = 0.79, N2 = 3.15) | | | | | | | |
| --- | --- | --- | --- | --- | --- | --- | --- |
| Preferred orientation. | trial1 | trial2 | trial3 | trial4 | trial5 | Mean. | SE |
| 0 [deg] | 0.003038 | 0.00217 | 0.005208 | 0.00434 | 0.003906 | 0.003733 | 0.000524 |
| 30 | 0.006076 | 0.013889 | 0.009549 | 0.009115 | 0.008681 | 0.009462 | 0.001261 |
| 60 | 0.11111 | 0.10938 | 0.10807 | 0.10026 | 0.10938 | 0.10764 | 0.001907 |
| 90 | 0.77604 | 0.75521 | 0.75564 | 0.76736 | 0.76823 | 0.7645 | 0.004001 |
| 120 | 0.095052 | 0.10677 | 0.11241 | 0.10677 | 0.10156 | 0.10451 | 0.002923 |
| 150 | 0.008681 | 0.012587 | 0.009115 | 0.012153 | 0.008247 | 0.010156 | 0.000917 |
|  |  |  |  |  |  |  |  |
|  |  |  |  |  |  |  |  |
| Table 5-8. N=4.72 (N1 = 1.57, N2 = 3.15) | | | | | | | |
| Preferred orientation. | trial1 | trial2 | trial3 | trial4 | trial5 | Mean. | SE |
| 0 [deg] | 0.008681 | 0.006076 | 0.005208 | 0.00651 | 0.006944 | 0.006684 | 0.000576 |
| 30 | 0.011719 | 0.012587 | 0.014323 | 0.013021 | 0.008681 | 0.012066 | 0.000945 |
| 60 | 0.13194 | 0.12413 | 0.11198 | 0.11762 | 0.12326 | 0.12179 | 0.00335 |
| 90 | 0.70747 | 0.73003 | 0.73568 | 0.73785 | 0.71267 | 0.72474 | 0.006179 |
| 120 | 0.1224 | 0.11372 | 0.11632 | 0.11068 | 0.13325 | 0.11927 | 0.003991 |
| 150 | 0.017795 | 0.013455 | 0.016493 | 0.014323 | 0.015191 | 0.015451 | 0.000772 |
|  |  |  |  |  |  |  |  |
|  |  |  |  |  |  |  |  |
| Table 5-9. N=5.51 (N1 = 2.36, N2 = 3.15) | | | | | | | |
| Preferred orientation. | trial1 | trial2 | trial3 | trial4 | trial5 | Mean. | SE |
| 0 [deg] | 0.014323 | 0.019097 | 0.014757 | 0.018229 | 0.017795 | 0.01684 | 0.000965 |
| 30 | 0.032552 | 0.029948 | 0.027778 | 0.022569 | 0.028212 | 0.028212 | 0.001641 |
| 60 | 0.15191 | 0.15191 | 0.16406 | 0.148 | 0.15495 | 0.15417 | 0.002708 |
| 90 | 0.61936 | 0.61068 | 0.60373 | 0.62457 | 0.60503 | 0.61267 | 0.004052 |
| 120 | 0.15408 | 0.16753 | 0.15451 | 0.15148 | 0.15929 | 0.15738 | 0.002835 |
| 150 | 0.027778 | 0.021267 | 0.035156 | 0.035156 | 0.034722 | 0.030816 | 0.002769 |
|  |  |  |  |  |  |  |  |

| Table 5-10. N=6.30 (N1 = 3.15, N2 = 3.15) | | | | | | | |
| --- | --- | --- | --- | --- | --- | --- | --- |
| Preferred orientation. | trial1 | trial2 | trial3 | trial4 | trial5 | Mean. | SE |
| 0 [deg] | 0.036024 | 0.028646 | 0.049913 | 0.033854 | 0.034722 | 0.036632 | 0.003549 |
| 30 | 0.063802 | 0.05816 | 0.067708 | 0.049479 | 0.043403 | 0.05651 | 0.004486 |
| 60 | 0.19184 | 0.19054 | 0.1875 | 0.19271 | 0.20182 | 0.19288 | 0.002403 |
| 90 | 0.46007 | 0.4579 | 0.45877 | 0.48307 | 0.44184 | 0.46033 | 0.006586 |
| 120 | 0.19705 | 0.19748 | 0.17665 | 0.18273 | 0.2053 | 0.19184 | 0.005262 |
| 150 | 0.051215 | 0.067274 | 0.059462 | 0.05816 | 0.072917 | 0.061806 | 0.00377 |
|  |  |  |  |  |  |  |  |
|  |  |  |  |  |  |  |  |
| Table 5-11. N=7.09 (N1=3.94, N2=3.15) | | | | | | | |
| Preferred orientation. | trial1 | trial2 | trial3 | trial4 | trial5 | Mean. | SE |
| 0 [deg] | 0.078559 | 0.067274 | 0.080729 | 0.073351 | 0.069878 | 0.073958 | 0.002537 |
| 30 | 0.10113 | 0.090278 | 0.11155 | 0.097656 | 0.078559 | 0.095833 | 0.005511 |
| 60 | 0.20703 | 0.2053 | 0.1849 | 0.19618 | 0.20747 | 0.20017 | 0.004335 |
| 90 | 0.31988 | 0.33724 | 0.34983 | 0.34418 | 0.30816 | 0.33186 | 0.007775 |
| 120 | 0.20312 | 0.18707 | 0.16884 | 0.19661 | 0.20703 | 0.19253 | 0.006821 |
| 150 | 0.090278 | 0.11285 | 0.10417 | 0.092014 | 0.12891 | 0.10564 | 0.007131 |
|  |  |  |  |  |  |  |  |
|  |  |  |  |  |  |  |  |
| Table 5-12. N=7.88 (N1 = 4.73, N2 = 3.15) | | | | | | | |
| Preferred orientation. | trial1 | trial2 | trial3 | trial4 | trial5 | Mean. | SE |
| 0 [deg] | 0.11068 | 0.11545 | 0.12977 | 0.10286 | 0.10634 | 0.11302 | 0.00469 |
| 30 | 0.1276 | 0.11849 | 0.13715 | 0.13759 | 0.10373 | 0.12491 | 0.006354 |
| 60 | 0.19184 | 0.1901 | 0.18056 | 0.19054 | 0.21267 | 0.19314 | 0.00528 |
| 90 | 0.24609 | 0.26302 | 0.26606 | 0.26606 | 0.23915 | 0.25608 | 0.005629 |
| 120 | 0.2079 | 0.17274 | 0.15321 | 0.17448 | 0.17752 | 0.17717 | 0.00879 |
| 150 | 0.11589 | 0.14019 | 0.13325 | 0.12847 | 0.16059 | 0.13568 | 0.007384 |
|  |  |  |  |  |  |  |  |

| Table 5-13. N=8.66 (N1 = 5.51, N2 = 3.15) | | | | | | | |
| --- | --- | --- | --- | --- | --- | --- | --- |
| Preferred orientation. | trial1 | trial2 | trial3 | trial4 | trial5 | Mean. | SE |
| 0 [deg] | 0.14063 | 0.13715 | 0.1645 | 0.12543 | 0.12543 | 0.13863 | 0.007154 |
| 30 | 0.14193 | 0.14453 | 0.15148 | 0.15061 | 0.125 | 0.14271 | 0.004779 |
| 60 | 0.18229 | 0.18663 | 0.16884 | 0.18837 | 0.19661 | 0.18455 | 0.004563 |
| 90 | 0.20312 | 0.22569 | 0.2322 | 0.22266 | 0.2105 | 0.21884 | 0.005276 |
| 120 | 0.19401 | 0.15234 | 0.13889 | 0.16189 | 0.15234 | 0.1599 | 0.009282 |
| 150 | 0.13802 | 0.15365 | 0.1441 | 0.15104 | 0.1901 | 0.15538 | 0.0091 |
|  |  |  |  |  |  |  |  |
|  |  |  |  |  |  |  |  |
| Table 5-14. N=9.45 (N1 = 6.30, N2 = 3.15) | | | | | | | |
| Preferred orientation. | trial1 | trial2 | trial3 | trial4 | trial5 | Mean. | SE |
| 0 [deg] | 0.15365 | 0.15061 | 0.17839 | 0.14583 | 0.14063 | 0.15382 | 0.006526 |
| 30 | 0.14757 | 0.15538 | 0.16233 | 0.16319 | 0.13759 | 0.15321 | 0.004814 |
| 60 | 0.18099 | 0.18533 | 0.16189 | 0.18316 | 0.19314 | 0.1809 | 0.005176 |
| 90 | 0.17578 | 0.19748 | 0.20616 | 0.19748 | 0.17969 | 0.19132 | 0.005801 |
| 120 | 0.19358 | 0.14974 | 0.13976 | 0.15712 | 0.15321 | 0.15868 | 0.009187 |
| 150 | 0.14844 | 0.16146 | 0.15148 | 0.15321 | 0.19575 | 0.16207 | 0.008692 |
|  |  |  |  |  |  |  |  |
|  |  |  |  |  |  |  |  |
| Table 5-15. N=10.24 (N1 = 7.09, N2 = 3.15) | | | | | | | |
| Preferred orientation. | trial1 | trial2 | trial3 | trial4 | trial5 | Mean. | SE |
| 0 [deg] | 0.16146 | 0.1697 | 0.18533 | 0.15234 | 0.15017 | 0.1638 | 0.006406 |
| 30 | 0.15061 | 0.15017 | 0.16493 | 0.16797 | 0.14323 | 0.15538 | 0.004729 |
| 60 | 0.18142 | 0.18186 | 0.15365 | 0.17491 | 0.19097 | 0.17656 | 0.006274 |
| 90 | 0.16102 | 0.1888 | 0.20443 | 0.1901 | 0.17361 | 0.18359 | 0.007459 |
| 120 | 0.19184 | 0.1467 | 0.13455 | 0.15712 | 0.14323 | 0.15469 | 0.009967 |
| 150 | 0.15365 | 0.16276 | 0.15712 | 0.15755 | 0.19878 | 0.16597 | 0.008331 |
|  |  |  |  |  |  |  |  |

| Table 5-16. N=11.13 (N1 = 7.88, N2 = 3.15) | | | | | | | |
| --- | --- | --- | --- | --- | --- | --- | --- |
| Preferred orientation. | trial1 | trial2 | trial3 | trial4 | trial5 | Mean. | SE |
| 0 [deg] | 0.1658 | 0.17188 | 0.19097 | 0.15582 | 0.15278 | 0.16745 | 0.006805 |
| 30 | 0.15799 | 0.15972 | 0.1671 | 0.17491 | 0.1428 | 0.1605 | 0.005347 |
| 60 | 0.17405 | 0.17839 | 0.15625 | 0.17057 | 0.19271 | 0.17439 | 0.005896 |
| 90 | 0.15625 | 0.17969 | 0.19531 | 0.18403 | 0.16753 | 0.17656 | 0.00675 |
| 120 | 0.1888 | 0.14236 | 0.13672 | 0.15365 | 0.1454 | 0.15339 | 0.009267 |
| 150 | 0.15712 | 0.16797 | 0.15365 | 0.16102 | 0.19878 | 0.16771 | 0.008125 |
|  |  |  |  |  |  |  |  |
|  |  |  |  |  |  |  |  |
| Table 5-17. N=14.96 (N1 = 11.81, N2 = 3.15) | | | | | | | |
| Preferred orientation. | trial1 | trial2 | trial3 | trial4 | trial5 | Mean. | SE |
| 0 [deg] | 0.16884 | 0.17882 | 0.1901 | 0.16363 | 0.15408 | 0.17109 | 0.00621 |
| 30 | 0.15538 | 0.15799 | 0.16797 | 0.17491 | 0.14757 | 0.16076 | 0.004811 |
| 60 | 0.17405 | 0.17448 | 0.15582 | 0.17231 | 0.18793 | 0.17292 | 0.005109 |
| 90 | 0.14931 | 0.17535 | 0.19444 | 0.17535 | 0.16319 | 0.17153 | 0.007475 |
| 120 | 0.19141 | 0.14453 | 0.13238 | 0.15321 | 0.14019 | 0.15234 | 0.010329 |
| 150 | 0.16102 | 0.16884 | 0.15929 | 0.16059 | 0.20703 | 0.17135 | 0.009076 |
|  |  |  |  |  |  |  |  |
|  |  |  |  |  |  |  |  |
| Table 5-18. N=18.9 (N1 = 15.75, N2 = 3.15) | | | | | | | |
| Preferred orientation. | trial1 | trial2 | trial3 | trial4 | trial5 | Mean. | SE |
| 0 [deg] | 0.16884 | 0.17882 | 0.19054 | 0.16363 | 0.15321 | 0.17101 | 0.006397 |
| 30 | 0.15538 | 0.15712 | 0.16797 | 0.17491 | 0.14844 | 0.16076 | 0.004724 |
| 60 | 0.17361 | 0.17491 | 0.15625 | 0.17231 | 0.18793 | 0.173 | 0.00504 |
| 90 | 0.15017 | 0.17535 | 0.19358 | 0.17535 | 0.16319 | 0.17153 | 0.007214 |
| 120 | 0.19097 | 0.14453 | 0.13325 | 0.15278 | 0.14019 | 0.15234 | 0.010163 |
| 150 | 0.16102 | 0.16927 | 0.15842 | 0.16102 | 0.20703 | 0.17135 | 0.009105 |
