# Supplementary material for: Development and Reorganization of Orientation Representation in the Cat Visual Cortex: Experience-Dependent Synaptic Rewiring in Early Life
Source: Front Neuroinform. 2020 Aug 20;14:41. doi: 10.3389/fninf.2020.00041 (PMC7468406; doi:10.3389/fninf.2020.00041)
Supplement: Supplementary file 10 [file Table_6.docx]

Supplementary Table 6-1. Numerical data of the average OSI at five trials of simulations under 12-orientation exposure. N: Total Monte Carlo steps of simulation.

| N | trial 1 | trial 2 | trial 3 | trial 4 | trial 5 | mean | SE |
| --- | --- | --- | --- | --- | --- | --- | --- |
| 0.00 | 0.33516 | 0.34268 | 0.33383 | 0.32488 | 0.33941 | 0.33519 | 0.003016 |
| 0.20 | 0.33065 | 0.34166 | 0.34524 | 0.33282 | 0.32378 | 0.33483 | 0.003866 |
| 0.39 | 0.34013 | 0.32738 | 0.33752 | 0.33409 | 0.32921 | 0.33367 | 0.00241 |
| 0.79 | 0.33156 | 0.33001 | 0.33333 | 0.33762 | 0.3269 | 0.33189 | 0.001781 |
| 1.58 | 0.38234 | 0.37028 | 0.3732 | 0.36592 | 0.37405 | 0.37316 | 0.002699 |
| 2.36 | 0.43655 | 0.43884 | 0.44495 | 0.4306 | 0.43717 | 0.43762 | 0.0023 |
| 3.15 | 0.47855 | 0.48117 | 0.49204 | 0.47602 | 0.43717 | 0.47299 | 0.009362 |
| 3.94 | 0.50428 | 0.50651 | 0.51547 | 0.49684 | 0.50308 | 0.50524 | 0.00302 |
| 4.73 | 0.5213 | 0.52177 | 0.52862 | 0.51157 | 0.51751 | 0.52016 | 0.002795 |
| 5.51 | 0.52986 | 0.52952 | 0.53764 | 0.52172 | 0.52534 | 0.52882 | 0.002664 |
| 6.30 | 0.53545 | 0.53584 | 0.5426 | 0.52533 | 0.53138 | 0.53412 | 0.002841 |
| 7.09 | 0.54019 | 0.53788 | 0.54577 | 0.52888 | 0.53341 | 0.53722 | 0.002885 |
| 7.88 | 0.54069 | 0.53892 | 0.54502 | 0.53041 | 0.53404 | 0.53782 | 0.002555 |
| 11.81 | 0.54221 | 0.5404 | 0.54626 | 0.53154 | 0.53437 | 0.53896 | 0.002665 |
| 15.75 | 0.54241 | 0.54041 | 0.54663 | 0.53142 | 0.53446 | 0.53907 | 0.002737 |

| Supplementary Table 6-2. Numerical data of the average OSI at five trials of simulations under single-orientation exposure following simulations under 12-orientation exposure. N1: Monte Carlo steps of simulation under 12-orientation exposure. N2: Monte Carlo steps of simulation under single-orientation exposure. N: Total Monte Carlo steps of simulation (N=N1+N2). | | | | | | | | |
| --- | --- | --- | --- | --- | --- | --- | --- | --- |
| N | N1, N2 | trial 1 | trial 2 | trial 3 | trial 4 | trial 5 | mean | SE |
| 0.79 | 0.00, 0.79 | 0.36273 | 0.35073 | 0.36591 | 0.38465 | 0.36005 | 0.36481 | 0.005567 |
| 1.58 | 0.00, 1.58 | 0.44728 | 0.44691 | 0.44446 | 0.46438 | 0.45202 | 0.45101 | 0.003559 |
| 2.36 | 0.00, 2.36 | 0.50394 | 0.50836 | 0.51174 | 0.50967 | 0.51211 | 0.50916 | 0.001475 |
| 3.15 | 0.00, 3.15 | 0.54412 | 0.53822 | 0.53564 | 0.54305 | 0.54147 | 0.5405 | 0.001571 |
| 3.35 | 0.20, 3.15 | 0.53823 | 0.53702 | 0.54105 | 0.53875 | 0.5414 | 0.53929 | 0.000839 |
| 3.54 | 0.39, 3.15 | 0.53811 | 0.53528 | 0.53681 | 0.53735 | 0.53614 | 0.53674 | 0.000488 |
| 3.94 | 0.79, 3.15 | 0.53328 | 0.5367 | 0.53565 | 0.53837 | 0.53771 | 0.53634 | 0.000893 |
| 4.73 | 1.58, 3.15 | 0.51476 | 0.52192 | 0.51844 | 0.5088 | 0.50872 | 0.51453 | 0.002613 |
| 5.51 | 2.36, 3.15 | 0.48971 | 0.4879 | 0.48765 | 0.48963 | 0.48228 | 0.48744 | 0.001357 |
| 6.30 | 3.15, 3.15 | 0.45411 | 0.4579 | 0.46714 | 0.46037 | 0.45745 | 0.4594 | 0.002178 |
| 7.09 | 3.94, 3.15 | 0.43535 | 0.44249 | 0.44877 | 0.43842 | 0.43808 | 0.44062 | 0.002335 |
| 7.88 | 4.73, 3.15 | 0.45023 | 0.44689 | 0.45369 | 0.44347 | 0.46937 | 0.45273 | 0.004495 |
| 8.66 | 5.51, 3.15 | 0.47515 | 0.4735 | 0.48219 | 0.46675 | 0.46937 | 0.47339 | 0.002653 |
| 9.45 | 6.30, 3.15 | 0.49592 | 0.49407 | 0.50191 | 0.49061 | 0.49219 | 0.49494 | 0.001958 |
| 10.24 | 7.09, 3.15 | 0.51742 | 0.51465 | 0.52099 | 0.51001 | 0.51079 | 0.51477 | 0.002053 |
| 11.03 | 7.88, 3.15 | 0.53043 | 0.52644 | 0.53243 | 0.51996 | 0.52168 | 0.52619 | 0.002409 |
| 14.96 | 11.81, 3.15 | 0.54249 | 0.54034 | 0.54619 | 0.53171 | 0.53413 | 0.53897 | 0.00267 |
| 18.90 | 15.75, 3.15 | 0.54241 | 0.54042 | 0.54663 | 0.53142 | 0.53446 | 0.53907 | 0.002737 |
